# Population-level impact of mass drug administration against schistosomiasis with anthelmintic drugs targeting juvenile schistosomes: a modelling study

**DOI:** 10.1016/j.lanmic.2024.101065

**Published:** 2025-05-21

**Authors:** Benjamin J Singer, Mireille Gomes, Jean T Coulibaly, Minoli Daigavane, Sophia T Tan, Isaac I Bogoch, Nathan C Lo

**Affiliations:** **Division of Infectious Diseases and Geographic Medicine, Department of Medicine, Stanford University, Stanford, CA, USA** (B J Singer DPhil, M Daigavane, S T Tan BA, N C Lo MD PhD); **Division of Epidemiology, School of Public Health, University of California, Berkeley, CA, USA** (B J Singer); **Ares Trading SA, an affiliate of Merck KGaA, Darmstadt, Germany** (M Gomes DPhil); **Unité de Formation et de Recherche Biosciences, Université Félix Houphouët-Boigny, Abidjan, Côte d’Ivoire** (J T Coulibaly PhD); **Centre Suisse de Recherches Scientifiques en Côte d’Ivoire, Abidjan, Côte d’Ivoire** (J T Coulibaly); **Swiss Tropical and Public Health Institute, Basel, Switzerland** (J T Coulibaly); **University of Basel, Basel, Switzerland** (J T Coulibaly); **Division of Infectious Diseases, Department of Medicine, University of Toronto, Toronto, ON, Canada** (I I Bogoc MD)

## Abstract

**Background:**

Schistosomiasis is a neglected disease caused by parasitic flatworms of the genus *Schistosoma* and affects more than 150 million people worldwide. Praziquantel, the drug used in public health control programmes, has minimal activity against juvenile schistosomes (within 6 weeks of infection) and imperfect cure rates. We aimed to model the population-level impact of hypothetical novel drug candidates, targeting both juvenile and adult schistosomes with various efficacies, across a range of baseline epidemiological settings.

**Methods:**

In this modelling study, we used a stochastic, individual-based mechanistic model of *Schistosoma mansoni* infection and simulated mass drug administration control programmes in diverse epidemiological environments. These programmes involved the administration, over a 5-year period at 75% coverage, of praziquantel (single-dose or two-dose regimens) or hypothetical novel drugs with various assumed efficacies against adult and juvenile schistosome parasites: novel drug A, with equivalent efficacy to praziquantel against adult schistosomes plus perfect (100%) efficacy against juvenile schistosomes; novel drug B, with higher efficacy than praziquantel against adult schistosomes and no activity against juveniles; and novel drug C, with higher efficacy than praziquantel against adult schistosomes plus perfect efficacy against juveniles. The outcomes were median observed *S mansoni* infection prevalence and infection intensity over time in simulated populations.

**Findings:**

In a simulated high-endemicity setting (baseline prevalence of *S mansoni* infection of 53%), modelled prevalence after a single treatment was 20·8% (uncertainty interval 15·8–23·6) for single-dose praziquantel, 17·8% (15·2–19·8) for two-dose praziquantel, 18·4% (13·4–21·4) for novel drug A, 16·0% (15·0–16·8) for novel drug B, and 13·4% (12·6–14·0) for novel drug C; at year 5, modelled prevalence was 14·6% (12·2–16·4) for single-dose praziquantel, 13·6% (11·6–14·6) for two-dose praziquantel, 11·8% (9·4–13·4) for novel drug A, 12·6% (11·6–13·4) for novel drug B, and 9·6% (9·0–10·4) for novel drug C. In a simulated low-endemicity setting (baseline prevalence 15%), modelled prevalence after a single treatment was 4·8% (3·6–5·8) for single-dose praziquantel, 4·2% (3·6–5·0) for two-dose praziquantel, 4·6% (3·2–5·4) for novel drug A, 4·0% (3·4–4·6) for novel drug B, and 3·6% (3·2–4·2) for novel drug C; at year 5, modelled prevalence was 3·0% (2·2–3·6) for single-dose praziquantel, 2·8% (2·2–3·4) for two-dose praziquantel, 2·6% (1·8–3·2) for novel drug A, 2·7% (2·2–3·2) for novel drug B, and 2·2% (1·8–2·6) for novel drug C.

**Interpretation:**

This study provides policy-relevant data that could help to guide the development and selection of novel drugs for schistosomiasis. Novel anthelmintic drugs that can kill both adult and juvenile schistosomes with higher efficacy than praziquantel could have some public health gains in control programmes for schistosomiasis, especially in high-burden settings. Novel drugs with increased efficacy against adult schistosomes are likely to have an initial and larger impact on disease control, whereas targeting juveniles could moderately improve longer-term control outcomes.

**Funding:**

US National Institutes of Health.

## Introduction

Schistosomiasis, a neglected disease that affects more than 150 million people globally, is caused by infection with parasitic flatworms of the *Schistosoma* genus.^1,2^ Schistosomes have a complex lifecycle that includes stages in fresh water, a snail intermediate host, and the human definitive host.^3^ Schistosomiasis is endemic across many low-income and middle-income countries, with the highest disease burden in Africa.^1,2^ The clinical presentation and sequelae of chronic schistosomiasis are species-specific. For intestinal schistosomiasis—caused by *Schistosoma mansoni* and *Schistosoma japonicum*—symptoms include abdominal pain, haematochezia, and fatigue, and chronic infection can result in complications of anaemia, periportal fibrosis, portal hypertension, pulmonary hypertension, and death.^3–5^

The drug praziquantel is the standard treatment for schistosomiasis and is recommended by WHO—alongside snail control and water, sanitation, and hygiene interventions—for use in public health control programmes; however, praziquantel has key limitations.^6–8^ WHO guidelines recommend annual preventive chemotherapy via mass drug administration (MDA) with single-dose praziquantel, aiming for at least 75% treatment coverage in all people aged at least 2 years in communities in which observed prevalence by stool or urine microscopy exceeds 10%.^8^ Praziquantel kills a large proportion of adult schistosomes, thereby reducing the production of eggs, which cause the immune-mediated tissue pathology and perpetuate transmission via their excretion in faeces or urine.^3–5,9,10^ At submicromolar concentrations, praziquantel activates transient receptor potential melastatin ion channels in adult schistosomes, inducing worm paralysis.^11^ Notably, praziquantel has little effectiveness against juvenile (immature) schistosomes—the form of the parasite until approximately 6 weeks after entry into a human host.^3,12,13^ As such, people who have been infected within this approximate timeframe will not be cured with a single praziquantel treatment if juvenile worms are present at the time of treatment.^12–15^ Furthermore, despite a relatively high dose of praziquantel (40 mg/kg), the cure rate in moderate-intensity and heavy-intensity infections (ie, those involving many schistosomes) can be low, due to imperfect efficacy of the drug against adult schistosomes.^16,17^ These observations have led some public health leaders and scientists to suggest that novel drugs—including those that have higher efficacy than praziquantel against adult schistosomes and can kill juvenile schistosomes—are necessary to reach WHO’s goals for the elimination of schistosomiasis as a public health problem,^18^ encouraging interest in drug development.^18–21^ Although some artemisinin-derivative drugs have been shown to have efficacy against juvenile schistosomes, there is interest in developing alternatives because artemisinin-derivative drugs are also used against malaria, and their widespread use in areas that are co-endemic for both schistosomiasis and malaria could lead to increased resistance in malaria parasites.^22,23^

Treatment-resistant juvenile schistosomes that cannot be killed by praziquantel could sustain transmission in endemic communities that receive annual MDA. Killing juvenile schistosomes in humans effectively delays the re-emergence of egg-patent infection, which is relevant to transmission, by approximately 6 weeks.^3,13^ Targeting juvenile schistosomes with novel drugs will therefore have a greater impact on infection prevalence and intensity in the weeks immediately after treatment than will drugs that kill only adult schistosomes. Existing knowledge of the effect of killing juvenile schistosomes comes from clinical trials of artemisinin-derivative drugs, most of which have been conducted in settings with exceptionally high transmission (>50% prevalence and a mean intensity of infection of >100 eggs per g faeces [EPG]) and short follow-up observations of 4–10 weeks.^22,24–27^ Although juvenile schistosome survival could be a barrier to the elimination of schistosomiasis, the effects of targeting juveniles with novel drugs in settings with different endemicities and over long time periods are poorly understood, as is the role of treatment-resistant juveniles in sustaining transmission in communities receiving MDA.

We modelled the impact of treatment-resistant juvenile schistosomes on the transmission dynamics of *S mansoni* and the impact of MDA in environments with various levels of transmission and epidemiological assumptions. Because mathematical models have been used to predict the long-term impact of related interventions against schistosomiasis,^28–34^ we simulated the use of hypothetical novel drug candidates targeting both juvenile and adult schistosomes with various efficacies to understand how alternative drugs could improve long-term public health control.

## Methods

### Data

For this modelling study, we used data on the prevalence and intensity of *S mansoni* infection from baseline surveys conducted as part of randomised trials by the Schistosomiasis Consortium for Operational Research and Evaluation.^35–37^ We identified communities in 75 study sites in Côte d’Ivoire with low (15 ± 2% prevalence), moderate (30 ± 2% prevalence), and high (50 ± 5% prevalence) endemicity to inform representative estimates for three baseline epidemiological environments in the model (appendix p 3). We used secondary datasets of individual-level data on *S mansoni* infection without direct identifiers, approved for use by the institutional review board at Stanford University (IRB protocol 71523).

### Model structure

We developed a stochastic, individual-based model, informed by previous models of schistosomiasis^28–33^ but with novel elements, to simulate infection with *S mansoni*. In particular, our model introduces an explicit representation of juvenile schistosomes. Each individual person in the model can be infected with a given number of juvenile (immature) schistosomes and adult schistosomes, with juveniles ageing into adults exactly 6 weeks after introduction.^3,12,13^ We generated a population of 500 people and assigned each person an age group (preschool child [aged 2–4 years], school-age child [aged 5–14 years], or adolescent or adult [aged ≥15 years]) and an individual-level susceptibility to infection (reflecting variation in behavioural, immunological, and other biological factors), which drove differences in worm burden. Infection intensity follows a heavy-tailed distribution, so we assumed that individual susceptibility β_*i*_ for an individual *i* in setting *s* and age group *a* is drawn from a log-normal distribution, $\beta_{i}\sim \mathrm{lognormal}(\mu_{s,a},\sigma_{s,a}^{2})$, where μ_*s,a*_ is the logarithm of the scale parameter, with the median susceptibility equalling exp(μ_*s,a*_) and σ_*s,a*_ determines the variance and positive skew. These parameters are calibrated to infection data from setting *s* and age group *a* (see Model calibration and validation). In each week-long time step, each individual *i* can be infected with a number of juvenile schistosomes drawn from a truncated negative binomial distribution with mean λ_all_β_*i*_ and overdispersion parameter *k*. This distribution models overdispersion in concentrations of cercaria infecting the human host,^38^ creating variation in the intensity of exposure events (including no exposure). In the base analysis, λ_all_ was set to 1 for model identifiability. Each adult worm has an average lifespan of 4 years.^39^ Infection and worm death are stochastic, whereas the maturation of juveniles into adults is deterministic to precisely isolate the effect of the juvenile life stage; we vary this maturation period in sensitivity analyses. Adult worm pairs generate eggs that are released in faeces according to a density-dependent fecundity function (appendix p 2). We set the EPG produced by a single worm pair to 4 EPG; this number was based on available literature and informed by model calibration.^40^

We implemented three versions of the model accounting for different assumptions on transmission dynamics. The first was a static model assuming a fixed force of infection (λ_all_), meaning the force of infection due to infected snails releasing cercariae is independent of human infection—this scenario broadly represents a large environmental reservoir of infection with contribution from numerous nearby communities. The second was a dynamic model assuming that the force of infection is proportional to mean human egg shedding 4–8 weeks earlier (to account for the lifecycle of schistosomes)—this scenario represents an environmental reservoir of infection that is supplied entirely by the local population.^3^ The third was a semi-dynamic model, assuming a 50% contribution to transmission from each of the static and dynamic models.

### Intervention simulation

We simulated MDA as a treatment for the population over a 5-year period. The base case assumption was 75% coverage randomly selected from 90% of the population, with the remaining 10% systematically missed or declining treatment.^8,41^ We compared five drug and dosing combinations: single-dose praziquantel (40 mg/kg); two doses of praziquantel (40 mg/kg) spaced 6 weeks apart; single-dose novel drug A, with equal efficacy to praziquantel against adult schistosomes and perfect (100%) efficacy against juvenile schistosomes; single-dose novel drug B, with near-perfect (99·9% parasite clearance) efficacy against adult schistosomes and no activity against juvenile schistosomes; and single-dose novel drug C, with near-perfect efficacy against adult schistosomes and perfect efficacy against juvenile schistosomes.

We simulated single-dose treatment as instantaneous parasite reduction, following a beta-binomial distribution with mean α and overdispersion parameter κ, separately for adult and juvenile schistosomes. We modelled the beta-binomial distribution by first drawing individual efficacy α_*i*_ from a beta distribution and inputting this into a binomial distribution. For two-dose treatment, the second parasite reduction step uses the same efficacy α_*i*_. For praziquantel, worm reduction applies only to adult schistosomes; for the novel drugs, worm reduction applies to both adult and juvenile schistosomes in some cases, although with different efficacies. The parasite reduction parameter α and overdispersion parameter κ are calibrated according to observed literature on intensity-stratified cure rate and overall egg reduction for single-dose praziquantel and two-dose praziquantel regimens (appendix pp 3–4).^16,17^

The study outcomes were median observed *S mansoni* infection prevalence and infection intensity over simulated populations after the modelled five drug regimens. We accounted for imperfect diagnostic sensitivity based on a model of three-sample duplicate Kato–Katz faecal microscopy, with sensitivity dependent on infection intensity and assuming perfect specificity.^42,43^

### Model calibration and validation

We calibrated our model to observed data on baseline epidemiology for each setting and treatment outcomes, using a grid search of the parameter space with an objective function of the sum of squared errors. We calibrated the model to three epidemiological environments: low endemicity (15% prevalence, 8% moderate-intensity and heavy-intensity infections, and geometric mean intensity of infection of 16 EPG), moderate endemicity (31% prevalence, 15% moderate-intensity and heavy-intensity infections, and geometric mean intensity of infection of 23 EPG), and high endemicity (53% prevalence, 29% moderate-intensity and heavy-intensity infections, and geometric mean intensity of infection of 42 EPG; appendix p 3).

We used cross-sectional data on *S mansoni* infection from Côte d’Ivoire (see Data) calibrating to three outcomes: infection prevalence (as observed with three-sample duplicate Kato–Katz faecal microscopy), geometric mean of infection intensity (measured in EPG), and the percentage of moderate-intensity and heavy-intensity infection among infected individuals (defined as ≥100 EPG). Including both the geometric mean of infection intensity and the percentage of moderate-intensity and heavy-intensity infection expresses the skewness of the infection intensity distribution, with more skewed distributions having greater geometric mean intensity relative to the percentage of moderate-intensity and heavy-intensity infections. The observed data for these calibration targets are shown in the appendix (p 3). We calibrated two model parameters to define individual susceptibility—a scale parameter μ and a dispersion parameter σ—and calibrated age-stratified susceptibility to data on the relative prevalence of schistosomiasis in three age groups (appendix pp 3–4).^44^

We calibrated the parasite-reduction parameter α and the overdispersion parameter κ of the treatment model and the overdispersion parameter *k* of the infection (or reinfection) model to fit praziquantel egg reduction and intensity-dependent cure rate in single-dose and two-dose schedules, on the basis of a review of literature values (appendix pp 3–4).^16,17^ We used the high-endemicity setting as the baseline for these calibrations, because trials of praziquantel have typically been conducted in high-burden settings. We assume that doses are spaced 6 weeks apart for two-dose praziquantel, as per common clinical practice, with follow-up observations 4 weeks after each dose.

We validated our model against data on the short-term effect of juvenile-targeting artemisinin-derivative drugs in combination with praziquantel (appendix pp 4–6).^24^ We validated our long-term model prediction and transmission dynamics under repeated MDA with praziquantel against data from a longer-term randomised trial (appendix pp 6–7).^35^

### Sensitivity and uncertainty analysis

In uncertainty analyses, we accounted for model (structural), parameter, and stochastic uncertainty. Model uncertainty is uncertainty in structural features of the model, such as whether schistosome fecundity is density-dependent or local features of transmission dynamics. Parameter uncertainty is uncertainty in fixed or fitted inputs of the model, such as duration of the juvenile life stage. Stochastic uncertainty arises from the fact that any single realisation of our stochastic model in a finite population will differ from others owing to random chance.

We conducted one-way sensitivity analyses to evaluate the contribution of the following model parameters and assumptions to the results: praziquantel cure rate and egg reduction, treatment coverage, duration of juvenile schistosome life stage, worm fecundity, and different baseline epidemiology (appendix p 8).^3,8,40^ We evaluated four alternative dynamic transmission models: the first using infection prevalence instead of population egg intensity to determine force of infection, the second using a non-linear saturation function of population egg intensity to determine the force of infection, the third using a compartmental model of snail infection, and the fourth modelling seasonal transmission (appendix p 7). We evaluated the impact of a sanitation intervention concomitant with the first round of MDA that permanently reduces the force of infection by 10%. We generated uncertainty intervals (UIs) accounting for parameter uncertainty in the effectiveness of praziquantel treatment and stochastic uncertainty in the finite population size of 500 individuals, taking the first quartile of simulations in the higher-efficacy treatment scenario and the third quartile of simulations in the lower-efficacy treatment scenario (appendix p 4). Analytical code is available online^45^ and epidemiological data are available from a public repository.^35^

### Role of the funding source

The funder of this study had no role in the study design, data collection, data analysis, data interpretation, or writing of the report.

## Results

Applying data from baseline surveys conducted in Côte d’Ivoire, we calibrated our microsimulation model to three epidemiological settings: low endemicity, moderate endemicity, and high endemicity (table 1; appendix pp 3–4). The model calibration achieved an overall good fit to the observed data (appendix p 3) and the calibrated model matches the data on the effects of MDA over time in the model validation (appendix pp 5–7).

**Figure 1:**
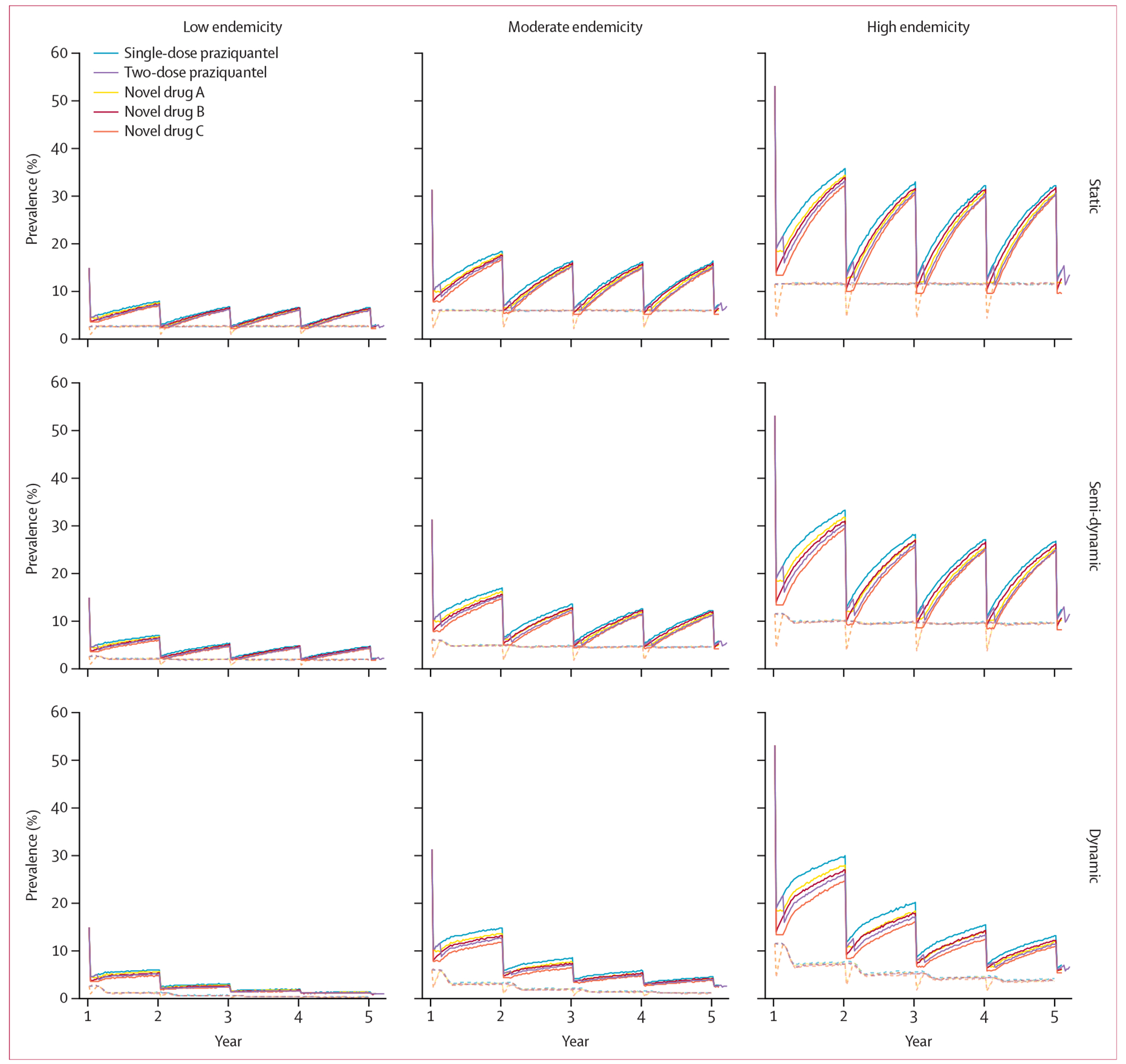
Comparison of the impact of MDA with different anthelmintic drugs and dosing schedules against *Schistosoma mansoni* under 75% treatment coverage under different epidemiological settings and transmission conditions Modelled infection prevalence over 5 years under low (baseline prevalence 15%; left), moderate (baseline prevalence 31%; middle), and high (baseline prevalence 53%; right) endemicity settings in the static (top), semi-dynamic (middle) and dynamic (bottom) models with the administration of single-dose praziquantel, two-dose praziquantel, novel drug A (the same efficacy against adult schistosomes as single-dose praziquantel plus perfect efficacy against juvenile schistosomes), novel drug B (near-perfect [99⋅9% parasite clearance] efficacy against adult schistosomes with no activity against juvenile schistosomes), or novel drug C (near-perfect efficacy against adult schistosomes plus perfect efficacy against juvenile schistosomes). The solid lines show the estimated prevalence of observed *S mansoni* infection with three-sample duplicate Kato–Katz faecal microscopy and the dashed lines show the estimated true prevalence of infection with juvenile schistosomes. We assumed 10% systematic non-adherence to MDA with 75% coverage. This analysis was repeated with alternative assumptions on treatment coverage and model structure (described in appendix p 8). MDA=mass drug administration.

The main analysis estimated the impact of providing single-dose praziquantel, two-dose praziquantel, novel drug A, novel drug B, or novel drug C, with 75% coverage of people aged at least 2 years, ina single MDA campaign (table 2) and over a 5-year period with annual MDA (figure 1).

In the simulated high-endemicity setting (baseline prevalence 53%) using the static model, estimated prevalence 4 weeks after completion of the first MDA was 20·8% (UI 15·8–23·6) for single-dose praziquantel, 17·8% (15·2–19·8) for two-dose praziquantel, 18·4% (13·4–21·4) for novel drug A, 16·0% (15·0–16·8) for novel drug B, and 13·4% (12·6–14·0) for novel drug C. At year 5, 4 weeks after the final round of treatment, prevalence was estimated at 14·6% (12·2–16·4) for single-dose praziquantel, 13·6% (11·6–14·6) for two-dose praziquantel, 11·8% (9·4–13·4) for novel drug A, 12·6% (11·6–13·4) for novel drug B, and 9·6% (9·0–10·4) for novel drug C. The prevalence of infection with juvenile schistosomes among people who test negative for *S mansoni* infection 4 weeks after completion of the first MDA was estimated as 11·5% (10·6–12·7) for single-dose praziquantel, 11·5% (10·6–12·7) for two-dose praziquantel, 2·9% (2·4–3·4) for novel drug A, 11·6% (10·6–12·7) for novel drug B, and 2·8% (2·4–3·4) for novel drug C. In the dynamic model, year 5 prevalence 4 weeks after the final round of treatment was 7·0% (5·6–8·2) for single-dose praziquantel, 6·2% (5·4–7·2) for two-dose praziquantel, 6·0% (5·0–7·0) for novel drug A, 6·2% (5·4–7·0) for novel drug B, and 5·4% (4·8–6·2) for novel drug C.

In the simulated low-endemicity setting (baseline prevalence 15%) using the static model, estimated prevalence 4 weeks after completion of the first MDA was 4·8% (UI 3·6–5·8) for single-dose praziquantel, 4·2% (3·6–5·0) for two-dose praziquantel, 4·6% (3·2–5·4) for novel drug A, 4·0% (3·4–4·6) for novel drug B, and 3·6% (3·2–4·2) for novel drug C. At year 5, 4 weeks after the final round of treatment, estimated prevalences were 3·0% (2·2–3·6) for single-dose praziquantel, 2·8% (2·2–3·4) for two-dose praziquantel, 2·6% (1·8–3·2) for novel drug A, 2·7% (2·2–3·2) for novel drug B, and 2·2% (1·8–2·6) for novel drug C (table 2, figure 1). The prevalence of infection with juvenile schistosomes among people who test negative for *S mansoni* infection 4 weeks after completion of the first MDA was 2·7% (2·2–3·2) for single-dose praziquantel, 2·7% (2·2–3·2) for two-dose praziquantel, 0·6% (0·4–0·9) for novel drug A, 2·7% (2·2–3·2) for novel drug B, and 0·6% (0·4–0·8) for novel drug C. In the dynamic model, year 5 prevalence 4 weeks after the final round of treatment was 1·0% (0·6–1·6) for single-dose praziquantel, 1·0% (0·6–1·4) for two-dose praziquantel, 1·0% (0·6–1·4) for novel drug A, 1·0% (0·6–1·4) for novel drug B, and 1·0% (0·6–1·2) for novel drug C. We repeated the analysis for a moderate-endemicity setting (baseline prevalence 31%) and conducted all analyses with a semi-dynamic model, with similar findings overall (figure 1).

**Figure 2:**
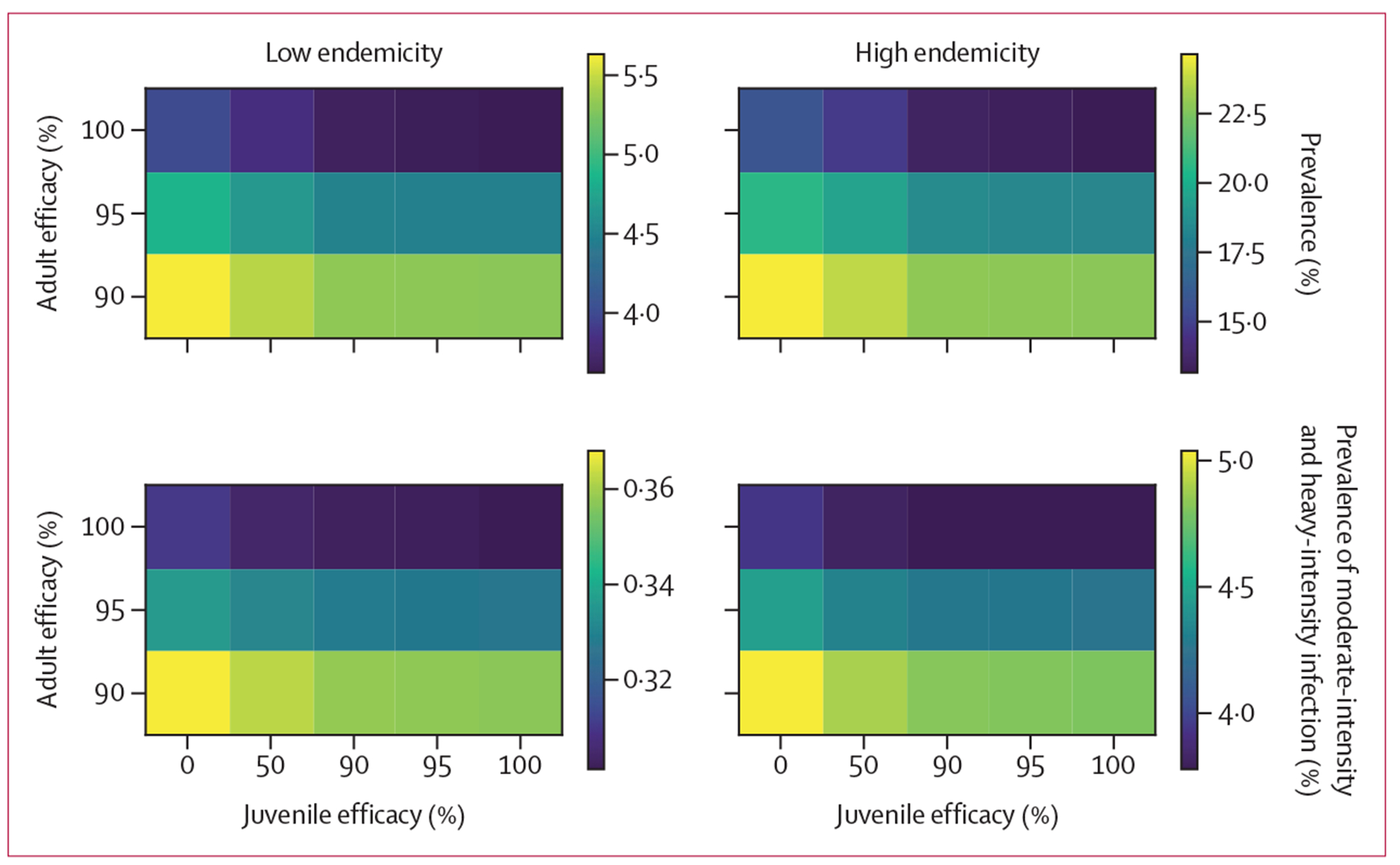
Comparison of overall population-level impact of novel drugs with different efficacies against adult and juvenile schistosomes after a single MDA treatment Using the static model, we compared novel drug candidates with various efficacies (parasite clearance percentage) against juvenile schistosomes (x-axis, 0–100%) and adult schistosomes (y-axis, 90–100%) in a single MDA treatment (4-week follow-up). In both low endemicity (left) and high endemicity (right) settings, we estimated mean *Schistosoma mansoni* infection prevalence (top) and the prevalence of moderate-intensity and heavy-intensity infections (bottom). As a reference, single-dose praziquantel has an efficacy against adult worms of 95% and has no activity against juvenile worms. MDA=mass drug administration.

**Figure 3:**
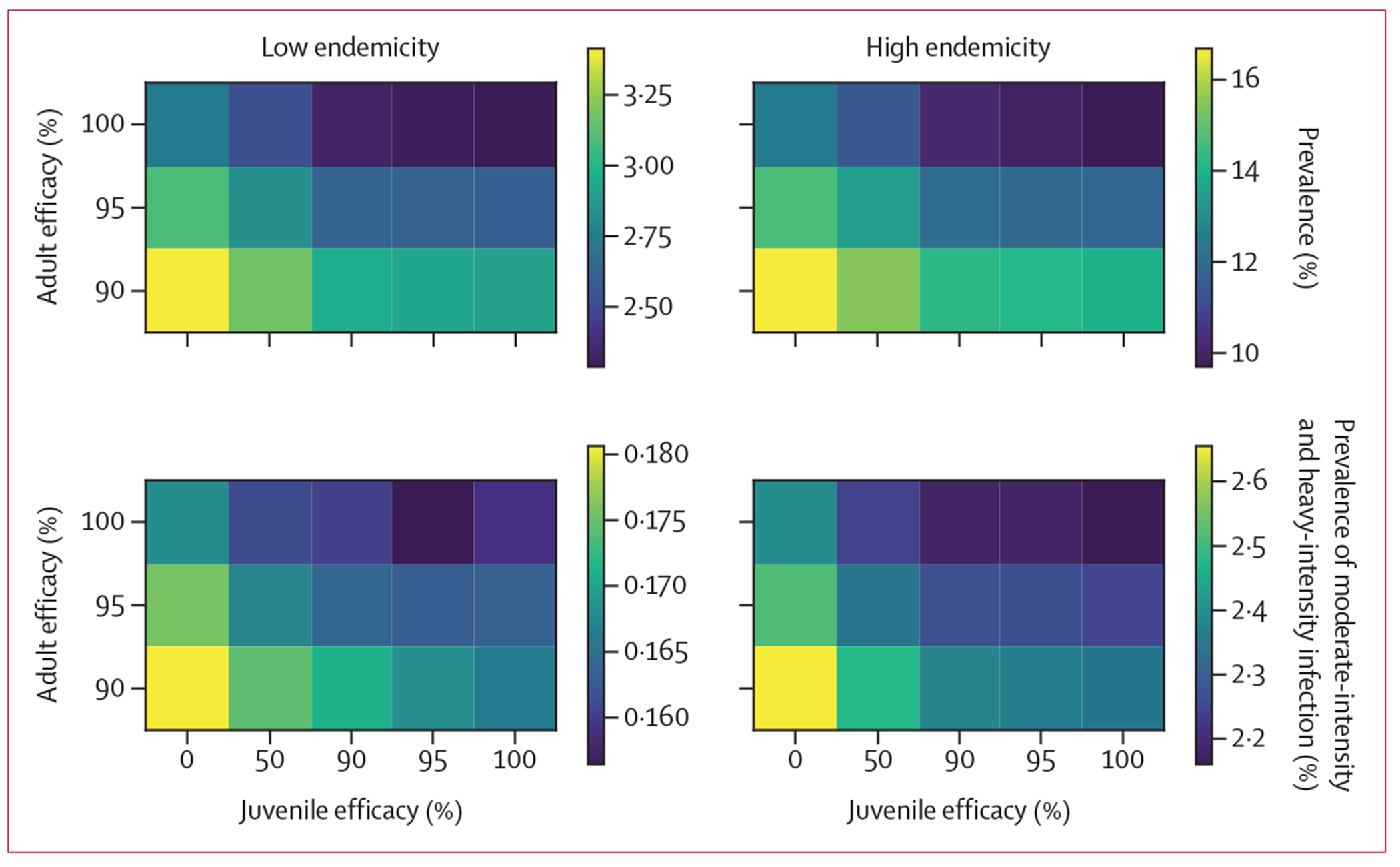
Comparison of overall population-level impact of novel drugs with different efficacies against adult and juvenile schistosomes at year 5 after annual MDA Using the static model, we compared novel drug candidates with various efficacies (parasite clearance percentage) against juvenile schistosomes (x-axis, 0–100%) and adult schistosomes (y-axis, 90–100%) in MDA programmes, estimating the outcome at year 5 after repeated annual MDA. In both low endemicity (left) and high endemicity (right) settings, we estimated mean *Schistosoma mansoni* infection prevalence (top) and the prevalence of moderate-intensity and heavy-intensity infections (bottom). Infections of this intensity are rare after repeated annual MDA, resulting in stochastic differences causing apparent non-monotonicity at low prevalences. As a reference, single-dose praziquantel has an efficacy against adult worms of 95% and has no activity against juvenile worms. MDA=mass drug administration.

We compared hypothetical novel drugs with various efficacies against adult and juvenile schistosomes after a single MDA round and after 5 years of annual treatment using a static model (figures 2, 3). Compared with single-dose praziquantel, after a single MDA round, increasing the efficacy against adult schistosomes yielded greater public health gains than adding activity against juvenile schistosomes (figure 2). Over a longer period (5-year follow-up), a drug with perfect efficacy against juvenile schistosomes had similar population-level benefits to a drug with increased efficacy against adult schistosomes (figure 3). We also evaluated scenarios with increased coverage of single-dose praziquantel, finding that a similar population-level benefit to a single round of MDA with novel drug A could be achieved by increasing coverage from 75% to 79% (appendix p 9). Similar population-level benefits to novel drugs B and C could be achieved by increasing the coverage of single-dose praziquantel to 85% and 89%, respectively.

We did multiple sensitivity analyses to evaluate the impact of single parameters and structural assumptions on the results of the model (appendix p 8). We reformulated the dynamic transmission model to include environmental saturation (ie, transmission is non-linear with community infection burden), to make transmission proportional to infection prevalence rather than worm burden, and to make transmission dependent on a compartmental model of snail infections; the results were similar to those of our main analysis (appendix pp 7–8, 14, 17–18). We implemented a seasonal model of transmission and found an advantage in targeting juvenile schistosomes in the high-transmission season, although the overall ranking of the drugs in terms of performance was similar to that in our main analysis (appendix pp 7, 15–16). We also varied treatment coverage from 60% to 90% and found that greater coverage led to larger reductions in infection prevalence, particularly with novel drugs B and C (appendix pp 8, 19–22); however, the ranking of the drugs remained the same as in the main analysis. We simulated a sanitation intervention concomitant with the first round of MDA, which led to larger prevalence reductions than MDA alone while maintaining the same ranking between drugs (appendix p 27). We varied the duration of the juvenile life stage between 4 weeks and 8 weeks; a longer juvenile stage increased the benefit of targeting juveniles (appendix pp 8, 25–26). Varying model parameters—egg production per worm pair (1–15 EPG per worm pair), praziquantel cure rate, and egg reduction—resulted in findings similar to those of the main analysis (appendix p 8).

## Discussion

Our individual-based simulation model of *S mansoni* infection accounting for juvenile schistosomes found that novel anthelmintic drugs that can kill both adult or juvenile schistosomes with higher efficacy than praziquantel could yield public health gains if used in the control of schistosomiasis. These benefits are greatest in high-transmission settings, assuming high treatment coverage, and could attenuate over time as prevalence is reduced. Our findings suggest that juvenile schistosomes have some role in the persistence of *Schistosoma* spp infections during repeated annual MDA, and that novel drugs with activity against juvenile schistosomes could therefore moderately improve long-term control efforts in some circumstances. These findings are supported by clinical trials studying the short-term effects of combinations of praziquantel with artemisinin compounds that have activity against juvenile schistosomes.^23–25^ However, by investigating a range of epidemiological settings and timescales, we show that increasing efficacy against adult schistosomes should also be prioritised during the development of novel drugs to maximise effects on public health. Praziquantel with repeat dosing could have similar public health effects to a novel drug with activity against juvenile schistosomes, although challenges of this strategy include a limited global supply of praziquantel, the requirement of resources for additional administration, and potential drug resistance. Future guidance on the development and selection of novel anthelmintics for schistosomiasis should prioritise both increasing efficacy against adult schistosomes and targeting juveniles to maximise sustained public health impact.

We find that praziquantel-resistant juvenile schistosomes have a moderate impact on the persistence of transmission during repeated MDA. Novel drugs targeting juvenile schistosomes (ie, the praziquantel-resistant life stage) delay the re-emergence of egg-patent infection in previously infected individuals by approximately 6 weeks, moderately reducing infection rates (and their subsequent contribution to transmission). This short juvenile period means that many years of repeated MDA are required before the impact is similar in magnitude to that of increased efficacy against adult schistosomes (if the true duration of juvenile resistance to praziquantel is longer than 6 weeks, this impact will be greater). Similar results have been found in a partially similar case of malaria, in which a modelling study found that the addition of drugs further targeting the sexual life stage of *Plasmodium* (ie, the late-stage gametocyte), which has implications for transmission, would have only a moderate benefit on the reduction of transmission.^46,47^ The overall impact of praziquantel-resistant juvenile schistosomes depended on the transmission setting and assumptions (eg, static *vs* dynamic model).

The findings from this study are supported by previous literature on the short-term effect of using artemisinin compounds in combination with praziquantel for the treatment of schistosomiasis.^23–26^ Artemisinin-derivative compounds are used as antimalarials, and have been shown to have efficacy against juvenile schistosomes.^48^ Because the endemic areas of schistosomiasis and malaria overlap, the use of artemisinin derivatives to control schistosomiasis could increase artemisinin resistance in *Plasmodium* spp.^49^ However, clinical trials of artemisinin-derivative compounds against schistosomiasis give insight into the short-term effects of targeting juvenile schistosomes in high-burden settings, showing that such targeting could improve outcomes from MDA.^24^ Our modelling study extends these insights to understand the potential long-term population impact of drugs with activity against juveniles across diverse epidemiological settings and under different assumptions regarding hypothetical novel drugs. We predict that targeting juveniles will have a smaller impact in settings with lower transmission intensity than in the exceptionally high-transmission settings studied in some clinical trials (appendix p 6).^24^

This study provides policy-relevant data to guide investigation into novel drugs as alternatives to praziquantel. A key uncertainty in the selection of drug candidates is the importance of activity against the juvenile life stage of schistosomes. Overall, our study supports the idea that novel drugs to treat *Schistosoma*, especially those with higher efficacy against adult schistosomes and activity against juvenile schistosomes, would have some public health impact in control programmes. Targeting juveniles could have greater impact in exceptionally high-transmission settings where the prevalence of juvenile schistosomes is highest from recent infection. We provide analyses to support the development of a target product profile to determine the ideal characteristics of a novel drug. Our study shows that, in future drug development, efficacy against juveniles should not be prioritised over increased efficacy against adult worms or the ease of achieving high coverage (eg, high pharmacological stability, low cost, and safety). In all cases, achieving high coverage of MDA is likely to maximise the relative impact of a novel drug compared with praziquantel. Additional considerations for novel anthelmintics could include activity against different *Schistosoma* species, a favourable pharmacokinetic profile, minimal side effects, and a high barrier to resistance.^18^ Although human schistosomes have not yet shown resistance to praziquantel, this remains a possibility given the increasing scale of MDA.

This study has limitations. The biology and transmission of *Schistosoma* spp is complex, and this modelling study made simplifying assumptions. Given the poorly characterised relationship between human infection, environmental reservoir, and transmission, we chose to conduct the analysis under a wide range of assumptions on transmission. We evaluated a static model (in which MDA did not affect transmission) and a fully dynamic model under various assumptions. We validated these models with data from a randomised trial on the short-term effect of praziquantel plus artemisinin compounds that treat juvenile schistosomes and with longer-term data from a randomised trial of MDA programmes with praziquantel (appendix pp 5–7). Although we show that our conclusions are robust to introducing a snail infection model, future studies could include more detailed snail models (such as a dynamic energy budget model) coupled with longitudinal data on snail and human infections.^50^ In regions with seasonal transmission of schistosomiasis, more individuals will have recent infections with juvenile schistosomes during the high-transmission season, meaning that the advantage of treatment that kills juveniles will increase during this season and will likewise decrease during the low-transmission season. The magnitude of these impacts will probably depend on the magnitude of seasonality of transmission, which varies greatly between schistosomiasis-endemic locations, with dependence on temperature and other factors.^51,52^

Although WHO guidelines recommend MDA (community-wide, for all individuals aged ≥2 years), some settings might still administer school-based preventive chemotherapy; in these settings, our results assuming lower population coverage are more relevant. Novel drugs with activity against juvenile schistosomes could be important for elimination efforts, although we did not evaluate elimination prospects in this study. Our study relied on literature estimates for cure rate, although such estimates could be biased downwards given the shedding of residual eggs from tissue and the conduct of such studies in high-transmission settings. We assumed that novel drugs would not have any effect on the viability of eggs already in tissue. We generated hypothetical parameters for novel drugs; as clinical trial data become available, these estimates can be updated to evaluate the effects of particular candidate drugs. Uncertainty remains regarding model inputs, such as the rate of egg production of a single worm pair, although we evaluated many of these inputs in sensitivity analysis with robust findings overall.^53,54^ Maturation and migration timings among juvenile schistosomes might differ. We did not test alternative timings for a second dose of praziquantel (eg, every 6 months). Finally, we used data only on *S mansoni*, although we expect our findings could be generalisable to other *Schistosoma* species.

In conclusion, MDA with a novel anthelmintic drug with higher efficacy than praziquantel against adult and juvenile schistosomes could yield public health gains against schistosomiasis. Development of novel drugs could improve prospects for the control and elimination of schistosomiasis, alongside expanding coverage and implementation of snail control and other preventive measures.

## Supplementary Material

1

## Figures and Tables

**Table 1:** Simulated population characteristics, baseline epidemiology, and model parameters for natural history and treatment

	Value
**Demographics**	

Population size, n	500
Age group	
Preschool children (2–4 years)	50 (10%)
School-age children (5–14 years)	125 (25%)
Adolescents or adults (≥15 years)	325 (65%)

**Natural history**	

Adult schistosome life expectancy, years^39^	4
Juvenile life stage duration, weeks^3,13^	6
Egg production per worm pair, EPG^40^	4
Maximum infection intensity, EPG	5000

**Treatment coverage**	

Treatment coverage	75%
Systematic non-adherence	10%

**Baseline epidemiology** ^35–37^	

Low-endemicity setting	
Prevalence	15%
Geometric mean intensity of infection, EPG	16 (3)
Proportion of moderate and heavy intensity infections	8%
Moderate-endemicity setting	
Prevalence	31%
Geometric mean intensity of infection, EPG	23 (4)
Proportion of moderate and heavy intensity infections	15%
High-endemicity setting	
Prevalence	53%
Geometric mean intensity of infection, EPG	42 (4)
Proportion of moderate and heavy intensity infections	29%

**Treatment** *	

Single-dose praziquantel	
Egg reduction	93%
Efficacy against juveniles	0%
Cure rate by intensity of infection	
Light	89%
Moderate	67%
Heavy	56%
Two-dose praziquante†	
Egg reduction	97%
Efficacy against juveniles	0%
Cure rate by intensity of infection	
Light	94%
Moderate	78%
Heavy	70%
Novel drug A‡	
Egg reduction	95%
Efficacy against juveniles	100%
Cure rate by intensity of infection	
Light	92%
Moderate	77%
Heavy	70%
Novel drug B§	
Egg reduction	98%
Efficacy against juveniles	0%
Cure rate by intensity of infection	
Light	97%
Moderate	87%
Heavy	81%
Novel drug C¶	
Egg reduction	100%
Efficacy against juveniles	100%
Cure rate by intensity of infection	
Light	100%
Moderate	99%
Heavy	99%

Data are n (%), %, or mean (SD), unless otherwise stated. Moderate-intensity and heavy-intensity infection is defined as infection intensity ≥100 EPG; reported SD are geometric SD. Epidemiological and treatment parameters are calibrated. EPG=eggs per g faeces.

* Egg reduction and cure rate assumes 4-week follow-up time in a high-endemicity setting, allowing for reinfection in a high-endemicity setting to reflect conditions in most praziquantel trials. These are calibrated model values; for literature values and references see appendix (p 3). Egg reduction is calculated on the basis of geometric mean EPG before and after treatment.

† Assumes doses are administered 6 weeks apart.

‡ Novel drug A assumes the same efficacy against adult schistosomes as single-dose praziquantel plus perfect efficacy against juvenile schistosomes.

§ Novel drug B assumes near-perfect (99·9% parasite clearance) efficacy against adult schistosomes with no activity against juvenile schistosomes.

¶ Novel drug C assumes near-perfect efficacy against adult schistosomes plus perfect efficacy against juvenile schistosomes.

**Table 2:** Public health impact of different anthelmintic drugs and dosing schedules on *Schistosoma mansoni* prevalence in different settings after a single MDA treatment

	Baseline	Single-dose praziquantel	Two-dose praziquantel	Novel drug A*	Novel drug B†	Novel drug C‡
**Prevalence, %**						
Low	14·6 (13·4–15·6)	4·8 (3·6–5·8)	4·2 (3·6–5·0)	4·6 (3·2–5·4)	4·0 (3·4–4·6)	3·6 (3·2–4·2)
Moderate	31·2 (29·8–32·6)	11·0 (8·4–12·6)	9·8 (8·2–11·0)	10·0 (7·4–11·8)	8·8 (8·2–9·6)	7·8 (7·2–8·6)
High	52·8 (51·2–54·4)	20·8 (15·8–23·6)	17·8 (15·2–19·8)	18·4 (13·4–21·4)	16·0 (15·0–16·8)	13·4 (12·6–14·0)

**Moderate-intensity and heavy-intensity infection prevalence, %**						
Low	1·2 (0·8–1·6)	0·4 (0·2–0·4)	0·2 (0·2–0·4)	0·2 (0·2–0·4)	0·2 (0·2–0·4)	0·2 (0·2–0·4)
Moderate	4·6 (3·8–5·2)	1·3 (0·8–1·6)	1·2 (0·8–1·6)	1·2 (0·8–1·6)	1·2 (0·8–1·4)	1·2 (0·8–1·4)
High	15·0 (14·0–16·2)	4·4 (3·4–5·3)	4·1 (3·4–4·8)	4·2 (3·3–5·1)	4·0 (3·4–4·5)	3·8 (3·2–4·3)

**Juvenile survival**§, %						
Low	—	2·7 (2·2–3·2)	2·7 (2·2–3·3)	0·6 (0·4–0·9)	2·7 (2·2–3·2)	0·6 (0·4–0·8)
Moderate	—	6·0 (5·3–6·8)	6·0 (5·2–6·8)	1·5 (1·2–1·9)	6·0 (5·3–6·8)	1·4 (1·2–1·8)
High	—	11·5 (10·6–12·7)	11·5 (10·6–12·7)	2·9 (2·4–3·4)	11·6 (10·6–12·7)	2·8 (2·4–3·4)

Data are median (UI; see Methods for details). Outcomes are measured 4 weeks after last dose administered in a single MDA campaign with 75% coverage, accounting for imperfect sensitivity of three-sample duplicate Kato–Katz faecal microscopy. Results are generated from the static model. Results from the dynamic model are identical owing to duration of juvenile life stage plus environmental life stages exceeding time to follow-up. UIs give the scale of stochastic variation and parameter uncertainty and are not a guide to the significance of differences between strategies. Parameter uncertainty in treatment effectiveness is not included for novel drugs B and C because these hypothetical drugs are determined by fixed assumptions (Methods, appendix p 4). MDA=mass drug administration. UI=uncertainty interval.

* Novel drug A assumes the same efficacy against adult schistosomes as single-dose praziquantel plus perfect efficacy against juvenile schistosomes.

† Novel drug B assumes near-perfect (99·9% parasite clearance) efficacy against adult schistosomes with no activity against juvenile schistosomes.

‡ Novel drug C assumes near-perfect efficacy against adult schistosomes plus perfect efficacy against juvenile schistosomes.

§ Juvenile survival is the proportion of cured individuals (ie, those who test positive for eggs at baseline but negative at follow-up) with surviving juveniles, which is calculated directly from the model with perfect accuracy (given the inability to test for juveniles).

## Data Availability

All data and analytical code for this study are available on GitHub (https://github.com/BenSinger01/Juvenile_Schistosomes).

## Research in context

### Evidence before this study

We searched PubMed for relevant articles published in English between database inception and May 22, 2024, using the search terms “schistosomiasis” OR “*Schistosoma*” together with “juvenile” OR “immature” AND “mass drug administration” OR “preventive chemotherapy” restricted to the title and abstract fields. This search identified 20 articles, including one randomised trial that found higher cure rates with praziquantel plus dihydroartemisinin–piperaquine (which has additional activity against juvenile schistosomes) than with praziquantel alone in patients infected with *Schistosoma mansoni* in a high-transmission environment with a short-term follow-up. We also included systematic reviews on drugs for the treatment of schistosomiasis, including artemisinin derivatives. We found no studies that evaluated the long-term, population-level effect, across diverse epidemiological environments, of mass drug administration programmes with novel drug combinations that are effective against juvenile as well as adult schistosomes.

### Added value of this study

We provide model-driven estimates of the population-level impact of novel drug candidates that target both juvenile and adult schistosomes with various efficacies, and compare these estimates with those for praziquantel alone in control programmes against schistosomiasis. Applying a simulation model of *S mansoni* infection, we find that mass administration (at 75% coverage) of novel drugs with higher efficacy than praziquantel against juvenile schistosomes, adult schistosomes, or both, shows some public health gains in control programmes for schistosomiasis, especially in high-burden settings. The benefits of novel drugs with activity against juvenile schistosomes are estimated to increase over time, owing to the compounded benefit of reduced infection burden and transmission, but higher efficacy against adult schistosomes yields larger initial impact. This study goes beyond previous work by estimating population-level outcomes to inform how these novel drugs could affect public health control and elimination goals.

### Implications of all the available evidence

Our findings suggest that novel drugs against *Schistosoma* with activity against juvenile schistosomes, higher efficacy than praziquantel against adult schistosomes, or both, are likely to have some population-level impacts in mass drug administration programmes. The benefits of such drugs are likely to be greatest in areas of high transmission and, potentially, in areas approaching elimination. These results could inform drug development decisions to align with the WHO global public health goal of eliminating schistosomiasis as a public health problem. Future work on drug development should aim for increased efficacy against adult and juvenile schistosomes, alongside ensuring safety, stability, and a high barrier to resistance. Treatment with any novel drug is likely to also require high treatment coverage, complementary interventions against the snail intermediate host, and water and sanitation measures to ensure the optimal public health control of schistosomiasis.
