# Supplementary material for: Population-level impact of mass drug administration against schistosomiasis with anthelmintic drugs targeting juvenile schistosomes: a modelling study
Source: Lancet Microbe. Author manuscript; Available in PMC 2025 Jul 10. (PMC12244469; doi:10.1016/j.lanmic.2024.101065)
Supplement: 1 [file NIHMS2094590-supplement-1.pdf]

THE LANCET
Microbe

Supplementary appendix

This appendix formed part of the original submission and has been peer reviewed.
We post it as supplied by the authors.

Supplement to: Singer BJ, Gomes M, Coulibaly JT, et al. Population-level impact of mass drug administration against schistosomiasis with anthelmintic drugs targeting juvenile schistosomes: a modelling study. *Lancet Microbe* 2025. https://doi.org/10.1016/j.lanmic.2024.101065

Table of Contents

Appendix

Technical Appendix
Figure A1: Relationship between infection intensity and sensitivity of the Kato-Katz technique using two slides each from three stool samples.
Table A1: Calibration of simulation model of schistosomiasis to low, moderate, and high endemicity settings and treatment.
Table A2: Values of calibrated parameters.
Table A3: Calibration to higher and lower efficacy scenarios for praziquantel.
Table A4: Values of calibrated parameters for higher and lower efficacy scenarios for praziquantel.
Table A5: Model validation comparing simulated and observed efficacy of praziquantel and dihydroartemisinin-piperaquine.
Table A6: Values of parameters for snail model.

Supplemental Tables and Figures

Table S1: Public health impact of single dose praziquantel after a single mass drug administration campaign at different coverage levels on *Schistosoma mansoni* prevalence.
Table S2: Comparison of the impact of different anthelmintic drugs and dosing schedules after a single mass drug administration campaign with 60% coverage on *Schistosoma mansoni* prevalence.
Table S3: Comparison of the impact of different anthelmintic drugs and dosing schedules after a single mass drug administration campaign with 90% coverage on *Schistosoma mansoni* prevalence.
Table S4: Comparison of the impact of different anthelmintic drugs and dosing schedules after a single mass drug administration campaign with 100% coverage on *Schistosoma mansoni* prevalence.

Figure S1: Model validation of long-term transmission dynamics with single dose praziquantel MDA in three epidemiologic settings.
Figure S2: Sensitivity analysis using snail model.
Figure S3: Sensitivity analysis using seasonal static model.
Figure S4: Sensitivity analysis using seasonal dynamic model.
Figure S5: Sensitivity analysis using a dynamic model with saturation.
Figure S6: Sensitivity analysis using a dynamic model based on prevalence.
Figure S7: Sensitivity analysis with 60% coverage.
Figure S8: Sensitivity analysis with 90% coverage.
Figure S9: Sensitivity analysis with 90% coverage and 0% nonadherence.
Figure S10: Sensitivity analysis with 100% coverage.
Figure S11: Sensitivity analysis with low schistosome fecundity.
Figure S12: Sensitivity analysis with high schistosome fecundity.
Figure S13: Sensitivity analysis with short juvenile life stage.
Figure S14: Sensitivity analysis with long juvenile life stage.
Figure S15: Sensitivity analysis with a WASH intervention.
Figure S16: Summary of sensitivity analysis on structural and parameter assumptions comparing single-dose praziquantel and novel drug A.
Figure S17: Year 5 static model outcomes before final mass drug administration with varying efficacy.
Figure S18: Year 5 dynamic model outcomes after final mass drug administration with varying efficacy.
Figure S19: Year 5 dynamic model outcomes before final mass drug administration with varying efficacy.

Appendix

This appendix provides additional methodological details on the study.

Technical Appendix

Density-dependent fecundity

To determine the eggs per gram of feces (EPG) for a given individual, which is dependent on the number of worm pairs, we used a density-dependent fecundity function. This is based on the idea that egg production decreases with increased number of worms and resource competition; this is a common feature of models of *Schistosoma* infection. The EPG for individual i is equal to:

$$E_{\max}\left(1-\exp\left(\frac{-CN_i}{E_{max}}\right)\right),$$

where N_i is the number of adult worm pairs (undergoing sexual reproduction) in individual (i), C is the number of eggs produced by a single worm pair (schistosome fecundity), and $E_{\max}$ is the maximum EPG. We set $E_{\max} = 5000$, approximately equal to the upper limit of observed data (1). We set $C = 4$ for our main analysis, based on available literature and informed by model calibration (2). Data on schistosome fecundity is limited, so we vary C in our sensitivity analyses (2–4). We implicitly assume stable stool output for an individual. This model simulates a declining per worm-pair contribution to EPG as the number of worms increases, to account for density-dependent fecundity.

Modeling imperfect diagnosis with Kato-Katz stool microscopy

We accounted for an imperfect observation process by modelling the intensity-dependent sensitivity of the Kato-Katz diagnostic method for schistosomiasis. We used observed data from the SCORE randomized trials in which persons were tested with Kato-Katz using three stool samples and two slides (3dx2s in Bärenbold et al.'s notation) (5), a methodology with greater sensitivity than Kato-Katz with fewer samples or slides. Most sampled individuals were in the 9–12 year age group. The sensitivity of Kato-Katz is nonlinearly related to the infection intensity (Figure A1), which has previously been characterized for the 3dx2s Kato-Katz methodology (according to Bärenbold et al.'s model). We use this model to stochastically simulate the observation process in our main analysis, in order to account for imperfect and infection intensity-dependent sensitivity. We assume perfect specificity of this diagnostic technique.

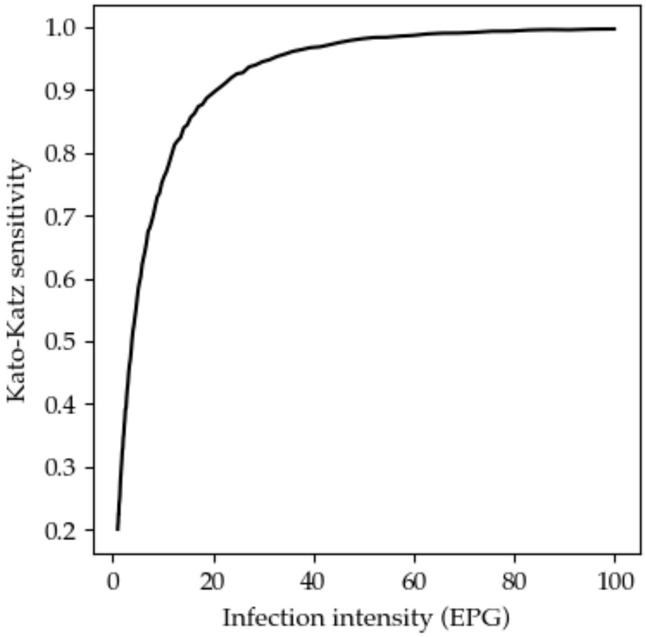

Figure A1: Relationship between infection intensity and sensitivity of the Kato-Katz technique using two slides each from three stool samples.

Model calibration
The model was calibrated as described in the Methods ('*Model calibration'* section). To calibrate to the observed epidemiologic data, we first identified communities within the SCORE dataset in Côte d'Ivoire that were 15±2% prevalence of infection (low endemic setting), 30±2% prevalence of infection (moderate endemic setting), or 50±5% prevalence of infection (high endemic setting). We then estimated the prevalence of moderate-to-heavy infection and geometric mean intensity of infection (GMI; measured in EPG or eggs per gram of feces). We calibrated the model to three outcomes: infection prevalence (measured assuming three stool samples and duplicate Kato-Katz), geometric mean of infection intensity (measured in eggs per gram of feces), and percent moderate and heavy infection intensity among infected individuals (defined as ≥ 100 eggs per gram of feces). Arithmetic mean is also reported in descriptive data. The chosen model calibration was closest to the observed data. The calibration targets are calculated from data gathered using the 3dx2s Kato-Katz methodology. The model obtained a good fit to the observed epidemiologic data (Table A1). After, we calibrated the model to observed data on single-dose and two-dose praziquantel, using egg reduction and intensity-specific cure rate; we obtained a good fit (Table A1).

We calibrated age-stratified susceptibility to data on relative prevalence of schistosomiasis in three age groups (6). Starting with overall scale and dispersion parameters of the susceptibility distribution calibrated to a the low endemicity setting, we introduced two parameters $r_{\text{preschool}}$ and r_{adult} to define the ratio of the mean susceptibility of preschool children to school-aged children, and adults to school-aged children, keeping the overall mean susceptibility constant. We used the low endemicity setting for this calibration due to the generally low prevalences in the studies informing the relative prevalence of schistosomiasis in the three age groups.

Table A1: Calibration of simulation model of schistosomiasis to low, moderate, and high endemicity and treatment.

**Baseline epidemiology**[1,7–10]			**Observed**	**Model**
**Low endemicity setting**	Prevalence (%)		15	14
	Geometric mean intensity (EPG)*		16	16
	Proportion moderate and heavy infections (%)		9	8
	Ratio of prevalence in pre-school children to prevalence in school-age children (%)		63	63
	Ratio of prevalence in adults to prevalence in school-age children (%)		83	83
**Moderate endemicity setting**	Prevalence (%)		30	31
	Geometric mean intensity (EPG)*		22	23
	Proportion moderate and heavy infections (%)		14	15
**High endemicity setting**	Prevalence (%)		54	53
	Geometric mean intensity (EPG)*		40	42
	Proportion moderate and heavy infections (%)		32	29
**Treatment**				
**Praziquantel (1 dose)**[11–20]	Egg reduction (%)		97	93
	Cure rate by intensity of infection	Light	86	89
		Moderate	68	67
		Heavy	59	56
**Praziquantel (2 dose)**[12,17–19,21]	Egg reduction (%)		99	97
	Cure rate by intensity of infection	Light	94	92
		Moderate	84	77
		Heavy	72	70

EPG; eggs per gram of feces
Moderate-to-heavy infection defined as infections with intensity>100 EPG
*The arithmetic mean intensity (among infected individuals) in the model is 33 EPG, 50 EPG, and 94 EPG in the low, moderate, and high endemicity settings, respectively.

Table A2: Values of calibrated parameters.

Parameter	Setting	Value
**Susceptibility distribution scale (μ_s)**	Low	-7.0
	Moderate	-5.4
	High	-3.9
**Susceptibility distribution dispersion (σ_s^2)**	Low	2.5
	Moderate	2.4
	High	2.5
**Ratio of susceptibility in pre-school children to susceptibility in school-age children ($r_{preschool}$)**	All	0.61
**Ratio of susceptibility in adults to susceptibility in school-age children (r_{adult})**	All	0.82
**Infection overdispersion (k)**	All	0.0175
**Praziquantel worm reduction (α)**	All	0.95
**Praziquantel overdispersion (κ)**	All	0.49

Model uncertainty

Our study has several sources of uncertainty. To explore model and parameter uncertainty, we include a variety of sensitivity analyses with different model assumptions (see Supplementary Tables and Figures). We present uncertainty intervals (UIs) to account for treatment parameter uncertainty and stochastic uncertainty and present these UIs alongside the results of our primary model. To incorporate parameter uncertainty into this interval, we re-ran our model of praziquantel treatment and novel drug A calibrated to an upper and lower estimate for the efficacy of praziquantel. These are informed by values found in the literature on single-dose praziquantel egg reduction and intensity-specific cure rate (11–20), and for two-dose praziquantel (12,17–19,21). We did not include any parameter uncertainty for novel drug B or novel drug C, since the characteristics of these hypothetical drugs are fixed assumptions without a relationship to existing drugs. The calibration targets and results are shown in Table A2, with some variation in quality of fit. To incorporate stochastic uncertainty, we simulated a community size of 500 individuals and noted the quartiles of simulated outcomes in these communities. To generate our final uncertainty interval, we defined the lower bound of the interval as the first quartile of outcomes from the higher praziquantel efficacy scenario, and the upper bound of the interval as the third quartile of outcomes from the lower praziquantel efficacy scenario. Note that the outcomes we measure (e.g., prevalence) are larger in the lower efficacy scenario and smaller in the higher efficacy scenario.

Table A3: Calibration to higher and lower efficacy scenarios for praziquantel.

				Observed	Model
**Higher efficacy praziquantel scenario**	**1 dose** (11–20)	Egg reduction (%)		99	97
		Cure rate by intensity of infection	Light	98	95
			Moderate	85	84
			Heavy	75	76
	**2 dose** (12,17–19,21)	Egg reduction (%)		99	98
		Cure rate by intensity of infection	Light	98	96
			Moderate	95	86
			Heavy	90	79
**Lower efficacy praziquantel scenario**	**1 dose** (11–20)	Egg reduction (%)		85	91
		Cure rate by intensity of infection	Light	75	86
			Moderate	60	60
			Heavy	50	48
	**2 dose** (12,17–19,21)	Egg reduction (%)		90	96
		Cure rate by intensity of infection	Light	93	92
			Moderate	80	75
			Heavy	70	64

Table A4: Values of calibrated parameters for higher and lower efficacy scenarios for praziquantel.

Scenario	Parameter	Value
**Higher efficacy praziquantel scenario**	Praziquantel worm reduction (α)	0.991
	Praziquantel overdispersion (κ)	0.54
**Lower efficacy praziquantel scenario**	Praziquantel worm reduction (α)	0.929
	Praziquantel overdispersion (κ)	0.5

Validation of modeling approach using data on artemisinin-derivative drug efficacy

We evaluated our model against data on the short-term effects of artemisinin-derivative drugs (which have activity juvenile schistosomes) combined with praziquantel for treatment of schistosomiasis to perform a validation of our

modeling approach. We performed a literature search for studies administering artemisinin-derivative drugs (which are the main drug with activity against juvenile schistosomes) against *S. mansoni* infections in combination with praziquantel which included a comparison to praziquantel alone and reported cure rates stratified by infection intensity. These criteria ensured that we would be able to assess the fit of our model to the data. We evaluated two systematic review articles from 2012 and 2016 on artemisinin-derivative drugs against schistosomiasis although the included studies did not include the requisite data (22–24). We performed a search on PubMed of papers published since 2016, and found one study that met our criteria, published in *PLOS Neglected Tropical Diseases* by Mnkugwe et al. in 2020 (25). This study compares the efficacy of praziquantel (PZQ) and a combination therapy of praziquantel and dihydroartemisinin-piperaquine (PZQ+DHP) in a very high transmission setting in Nyamikoma, a village in Tanzania on the shores of Lake Victoria. In this study, 639 school children testing positive for *S. mansoni* were randomly assigned to control (praziquantel) or intervention (praziquantel with dihydroartemisin-piperaquine) groups at a 1:1 ratio. Observation of study outcomes occurred at 3 weeks and 8 weeks after treatment, using two-sample two-slide Kato Katz (5).

We calibrated the model and values of the susceptibility distribution parameters and infection overdispersion to fit the baseline epidemiologic characteristics from Mnkugwe et al. With this calibration, we found that our model gave results for the impact of the combination therapy (PZQ+DHP) within confidence intervals calculated from Mnkugwe et al.'s results and sample sizes, for certain values of schistosome fecundity. We found that our predictions for the impact of single-dose praziquantel in the very high transmission setting of Mnkugwe et al.'s study differed somewhat from their results, with our predictions showing a larger variability of cure rate stratified by initial infection intensity but with similar overall cure rate. Most studies on praziquantel efficacy report a decline of cure rate with greater infection intensity, so we chose not to adjust our model to fit Mnkugwe et al.'s findings for praziquantel alone. We prioritized evaluation of this model validation at the 8 week follow up. This analysis informed our choice of schistosome fecundity and values of infection overdispersion for our main analysis. The validation is limited to a short-term follow up only, due to the short-term nature of most existing artemisinin-derivative drug trials for schistosomiasis. Overall, this analysis supports this modeling approach to estimating the impact of novel drug combinations for schistosomiasis.

Table A5: Model validation comparing simulated and observed efficacy of praziquantel and dihydroartemisinin-piperaquine.

				**Observed (95% CI)**	**Model simulation**
**Baseline**		Arithmetic mean intensity (EPG)		376 (350–440)*	**365**
		Proportion moderate and heavy infections (%)		72 (67–77)	**72**
**Treatment**					
**Praziquantel alone**	**3 week follow-up**	Egg reduction (%)		95 (93–97)	**95**
		Cure rate (%)		81 (77–85)	74
		Cure rate by intensity of infection	Light	83 (74–89)†	90
			Moderate	83 (76–88)†	73
			Heavy	78 (68–85)†	65
	**8 week follow-up**	Egg reduction (%)		88 (84–91)	92
		Cure rate (%)		64 (59–69)	**63**
		Cure rate by intensity of infection	Light	63 (53–73)†	86
			Moderate	67 (59–74)†	**61**
			Heavy	60 (50–69)†	**51**
**Praziquantel and dihydroartemisinin-piperaquine**	**3 week follow-up**	Egg reduction (%)		95 (93–98)	**96**
		Cure rate (%)		88 (84–91)	82
		Cure rate by intensity of infection	Light	94 (87–97)†	**93**
			Moderate	90 (83–94)†	**83**
			Heavy	81 (71–87)†	**76**
	**8 week follow-up**	Egg reduction (%)		94 (91–96)	**95**
		Cure rate (%)		82 (77–86)	**79**
		Cure rate by intensity of infection	Light	88 (79–93)†	**92**
			Moderate	82 (74–88)†	**80**
			Heavy	76 (66–84)†	**71**

*Calculated based on sample size assuming a lognormal distribution of arithmetic mean intensity
†Calculated based on sample size assuming a binomial distribution of cure rate
Other CIs are quoted directly from Mnkugwe et al.
Bolded values in the "Model" column are within the 95% confidence intervals for the observed value.

Note: We prioritized model validation at 8-week time point.

Discussion of the model validation using data on artemisinin-derivative drug efficacy and relation to main study

We re-calibrated our model to the epidemiologic setting found in Mnkugwe et al., and found the model results simulating the combination of praziquantel and dihydroartemisinin-piperaquine (PZQ+DHP) were similar to those observed in the study. Specifically, the model tested the short-term impact of PZQ+DHP – simulated via Novel Drug A, a drug with high activity against juveniles and activity against adults equal to praziquantel alone (therefore, similar to PZQ+DHP) – and found the model-estimated impact to be similar to the observed effect of PZQ+DHP. In particular, novel drug A demonstrated a moderate improvement compared to praziquantel alone in cure rate. However, in the main analysis under low, moderate, and high endemicity settings, we find a smaller impact of Novel Drug A compared to praziquantel. This is due to the difference in baseline epidemiology; the infection prevalence and intensity in the setting studied by Mnkugwe et al. far exceeded any of the settings included in our main analysis (using epidemiologic data from the SCORE trials). The rate of infection was much higher in the Mnkugwe et al. setting (based on the calibrated model) compared to the epidemiologic settings from the SCORE study which supplied the calibration data for our model, and which is thought to represent high transmission settings. The degree of benefit of drugs with anti-juvenile activity is related to the intensity of transmission. Overall, the SCORE study sites are likely to be more representative of endemic settings, and are themselves likely to be considered highly endemic.

In the setting studied by Mnkugwe et al., individuals cured by treatment with praziquantel are subject to reinfection at a very high rate—around 3% per week, according to our model. Contrariwise, in our high transmission setting (calibrated to SCORE settings) with 52% prevalence, only around 1% of cured individuals are reinfected each week after treatment. That is to say, the force of infection in our high transmission setting is roughly one third of that in the setting studied by Mnkugwe et al. This determines the relative benefit seen from drug activity against juveniles, since individuals cleared of juvenile worms will not see detectable infections reemerge for at least six weeks after treatment. Because the force of infection in our high transmission setting is substantially lower than in the setting studied by Mnkugwe et al., there are fewer recent infections with juvenile worms, reducing the relative benefit of drugs with activity against juveniles.

Additionally, the large benefits found for PZQ+DHP by Mnkugwe et al. may also be due to some increased efficacy against adult schistosomes compared to PZQ alone. The degree of DHP's efficacy against adults is uncertain, so we ignored the possibility of increased adult efficacy in our model of PZQ+DHP. There is however some evidence that artemisinin-derivative drugs have efficacy against adult schistosomes, with trials showing that egg patent infections can decrease in the short term using artemisinin-derivative drugs alone (23,26). Introducing adult efficacy would add an extra degree of freedom to our model, allowing worm fecundity or infection overdispersion to vary.

Another study on the effects of artemisinin-derivative drugs in combination with praziquantel by De Clercq et al. (26) also worked in a very high transmission setting (60% prevalence and arithmetic mean 160EPG), and found an odds ratio of egg-patent schistosomiasis between praziquantel and the combination therapy of 2.81. However, after 12 weeks this fell to 1.00, and after 24 weeks to 0.92. This supports our finding that, in the absence of additional rounds of mass drug administration, the relative benefit of drugs targeting juvenile schistosomes reduces over time compared to praziquantel alone.

Model validation of long-term transmission dynamics under repeated MDA

We performed an additional model validation. We compared our long-term model prediction and transmission dynamics under repeated MDA with praziquantel using data from the SCORE trials in Côte d'Ivoire. The dataset provided 5-year longitudinal prevalence data of single-dose praziquantel MDA in three treatment arms (Arm 1: every year, Arm 2: first two years, Arm 3: first and third years).

The model was calibrated to baseline cross-sectional data (not longitudinal data). Therefore, we can then compare the model projections against the observed longitudinal data by simulating the MDA strategy in each of the SCORE treatment arms, matching average reported coverage for each arm in each setting (low endemicity, moderate endemicity, and high endemicity). The model validation compares our projected model outcomes to observed longitudinal prevalence data for SCORE communities. To account for potential differences in transmission dynamics between communities included in the SCORE trials, we performed this validation with the static, semi-dynamic, and dynamic models. As shown in Figure S1, prevalence in at least one of our simulations lies within the

range reported in SCORE for most time points in each setting and study arm. This supports the model's ability to capture schistosomiasis dynamics over long-term MDA.

Dynamic transmission models
We implemented three models for *S. mansoni* infection with different structures and assumptions: static model, semi-dynamic model, and fully dynamic model. In the dynamic model, we assume that the force of infection multiplier λ_{all} at time t is dependent on the mean total egg burden in the population from time $t-4$ to $t-8$ weeks. This assumes transmission should be proportional to the infectious material contributed to the environmental reservoir (eggs excreted in feces, which later hatch into miracidia, enter snail, and emerge as infectious cercaria). This assumes excreted eggs have equal chance of entering the environmental reservoir. The time lag of 4-8 weeks is to account for the lag effect of the life cycle of *Schistosoma* within the environmental reservoir, which takes many weeks from egg shedding to maturation in the snail intermediate host to the production of infectious cercaria. In a given time step, the maximal infectious exposure was truncated at 100 cercaria. In our main analysis *S. mansoni* infection, we set the force of transmission multiplier as a function of time $\lambda_{all}(t)$ to:

$$\lambda_{all}(t) = \left(a + (1-a)\sum_{\tau=t-8}^{t-4}\sum_i E_i(\tau) \Big/ 5\sum_i E_i(0)\right),$$

where $E_i(\tau)$ is the egg intensity of individual i at time τ, where $\tau = 0$ at baseline, and a is a parameter determining the degree to which the current prevalence affects the force of transmission. For the *static* scenario, $a = 1$; for the *semi-dynamic* scenario, $a = 1/2$; and for the *dynamic* scenario, $a = 0$.

As sensitivity analyses, we evaluate alternative formulations of the dynamic transmission model. In the first sensitivity analysis, we evaluate including a saturation function (accounting for non-linear relationships between the population infection burden and transmission), modelling an environment that can only support a finite amount of transmission. In this case,

$$\lambda_{all}(t) = \left(a + (1-a)\left(1 - \exp\left(\frac{-\sum_{\tau=t-8}^{t-4}\sum_i E_i(\tau)}{5b}\right)\right)\Big/\left(1 - \exp\left(\frac{-\sum_i E_i(0)}{b}\right)\right)\right),$$

where b is a parameter which determines the strength of the saturation, with higher values of b corresponding to weaker saturation. This model approaches the dynamic model without saturation as b approaches infinity, and the purely static model as b approaches zero. We set b=10 to create strong saturation effects in the high endemicity setting and weak saturation effects in the low endemicity setting, based on values from model calibration.

In the second sensitivity analysis, we evaluate scenarios in which all infected individuals contribute equally to transmission, regardless of egg burden. In this case,

$$\lambda_{all}(t) = \left(a + (1-a)\sum_{\tau=t-8}^{t-4}\sum_i \operatorname{sgn}(E_i(\tau)) \Big/ 5\sum_i \operatorname{sgn}(E_i(0))\right),$$

where sgn is the sign function, which equals 1 for all positive numbers and 0 at zero.

We also performed a sensitivity analysis in which force of infection varies seasonally. In the static version:

$$\lambda_{all}(t) = 1 + \frac{1}{2}\sin\left(\frac{2\pi(t+\tau)}{52}\right),$$

so that the overall force of transmission parameter varies from 0.5 to 1.5, with a period of 52 weeks (ie, one year). The variable τ is used to determine whether treatment occurs in the low transmission season or the high transmission season. In the dynamic version of the seasonal model:

$$\lambda_{all}(t) = \left(1 + \frac{1}{2}\sin\left(\frac{2\pi(t+\tau)}{52}\right)\right) \cdot \sum_{\tau=t-8}^{t-4}\sum_i E_i(\tau) \Big/ 5\sum_i E_i(0),$$

where the dynamic transmission term is the same as in the fully dynamic version of the egg burden based model without saturation above.

Snail model
To demonstrate that our dynamic model is a valid simplification of the complex dynamics of *Schistosoma* infection, we implemented an explicit model of a snail reservoir for comparison. We used a deterministic compartmental SEI model structure informed by prior literature (27,28). We treated the miracidial and cercarial life stages of schistosomes as instantaneous, because they occur on a much smaller time scale than our model's time step of one week. Our snail model equations are as follows:

$$\frac{dS}{dt} = B_0\left(1 - \frac{S(t) + E(t) + I(t)}{K}\right)\big(S(t) + E(t)\big) - \eta G(t)S(t) - \mu S(t),$$

$$\frac{dE}{dt} = \eta G(t)S(t) - \rho E(t) - \mu E(t),$$

$$\frac{dI}{dt} = \rho E(t) - (\mu + \mu_I)I(t),$$

where $S(t)$is the density of susceptible snails, $E(t)$is the density of exposed (prepatent) snails, $I(t)$ is the density of infected snails, $G(t)$ is the sum of the human population's egg intensity in the time step, B_0 is the base birth rate of snails, K is the carrying capacity density of the environment, η is the snail infection rate as mediated by free miracidia, μ is the death rate of susceptible and exposed snails, ρ is the maturation rate of miracidia in snails, and μ_I is the differential death rate of infected snails. We approximated this model in our 1-week discrete time steps using the Euler method. We scaled the susceptibility for all members of the human population by $qI(t)$, where q is the human exposure to the snail reservoir as mediated by free cercariae. We set all parameters except η and q to the values given by Civitello et al. (27), and we calibrated η and q to the high transmission setting with 53% infection prevalence, 42EPG GMI, and 29% moderate-to-heavy infections, assuming no change in other parameters from the human-only model. The resulting parameter values are given in Table A6.

Table A6: Values of parameters for snail model

Parameter	Source	Value
$\boldsymbol{B_0}$	Civitello et al. 2022	0.7 $week^{-1}$
$\boldsymbol{K}$	Civitello et al. 2022	5 L^{-1}
$\boldsymbol{\mu}$	Civitello et al. 2022	0.07 $week^{-1}$
$\boldsymbol{\mu_I}$	Civitello et al. 2022	0.28 $week^{-1}$
$\boldsymbol{\rho}$	Civitello et al. 2022	0.25 $week^{-1}$
$\boldsymbol{\eta}$	Calibration	1.55×10^{-7} EPG^{-1}
$\boldsymbol{q}$	Calibration	23 L

Sensitivity analyses

To explore model and parameter uncertainty, we performed a range of sensitivity analyses. To explore transmission model uncertainty, we tested variations in the dynamic transmission model, including a snail model (Figure S2), a dynamic model with saturation (Figure S5), a dynamic model based on prevalence (Figure S6), and models with seasonality of transmission (Figures S3–4). To explore the effect of coverage we tested coverage levels from 60% to 100%, and variants with 0% and 10% systematic non-adherence to treatment (Figures S7–S10). To explore parameter uncertainty in schistosome fecundity we tested worm-pair fecundity of 1EPG and 15EPG (Figures S11–S12). To explore parameter uncertainty in juvenile life stage duration we tested durations of 4 and 8 weeks (Figures S13–S14).

Supplemental Tables and Figures

Table S1: Public health impact of single dose praziquantel after a single mass drug administration campaign at different coverage levels on *Schistosoma mansoni* prevalence.

	Low endemicity		Moderate endemicity		High endemicity	
**Coverage (%)**	**Prevalence**	**Moderate and heavy prevalence**	**Prevalence**	**Moderate and heavy prevalence**	**Prevalence**	**Moderate and heavy prevalence**
**77**	4.6	0.2	10.4	1.2	19.8	4.1
**78**	4.4	0.2	10.2	1.2	19.2	4.0
**79**[a]	4.2	0.2	9.8	1.0	18.8	3.8
**80**	4.2	0.2	9.6	1.0	18.4	3.7
**81**	4.0	0.2	9.4	1.0	18.0	3.6
**82**	3.8	0.2	9.2	1.0	17.6	3.4
**83**	3.8	0.2	8.8	1.0	17.2	3.3
**84**	3.6	0.2	8.6	0.8	16.8	3.1
**85**[b]	3.4	0.2	8.4	0.8	16.2	3.0
**86**	3.4	0.2	8.0	0.8	15.8	2.8
**87**	3.2	0.2	7.8	0.8	15.4	2.7
**88**	3.0	0.2	7.4	0.6	15.0	2.6
**89**[c]	3.0	0.2	7.2	0.6	14.6	2.4
**90**	2.8	0.2	7.0	0.6	14.2	2.2

This analysis was performed to find the coverage of single-dose praziquantel MDA closest (in terms of median treatment outcomes) to the hypothetical novel drugs administered at 75% coverage, assuming 10% systematic nonadherence.

[a] Closest outcomes to novel drug A.
[b] Closest outcomes to novel drug B.
[c] Closest outcomes to novel drug C.

Table S2: Comparison of the impact of different anthelmintic drugs and dosing schedules after a single mass drug administration campaign with 60% coverage on *Schistosoma mansoni* prevalence.

	Setting	Baseline	1-dose praziquantel	2-dose praziquantel	Novel drug A	Novel drug B	Novel drug C
**Prevalence (%)**	Low	14.6 (13.4–15.6)	6.8 (5.6–7.8)	6.4 (5.6–7.2)	6.4 (5.2–7.5)	6.2 (5.4–6.8)	5.8 (5.2–6.4)
	Moderate	31.2 (29.8–32.6)	15.0 (12.6–16.8)	14.0 (12.6–15.4)	14.4 (12.0–16.0)	13.4 (12.4–14.4)	12.6 (11.6–13.4)
	High	52.8 (51.2–54.4)	27.2 (23.0–29.8)	25.0 (22.6–26.8)	25.4 (21.0–28.0)	23.4 (22.4–24.4)	21.4 (20.4–22.2)
**Moderate and heavy infection prevalence (%)**	Low	1.2 (0.8–1.6)	0.4 (0.2–0.8)	0.4 (0.2–0.6)	0.4 (0.2–0.6)	0.4 (0.2–0.6)	0.4 (0.2–0.6)
	Moderate	4.6 (3.8–5.2)	1.9 (1.4–2.4)	1.8 (1.4–2.2)	1.8 (1.4–2.3)	1.8 (1.4–2.2)	1.8 (1.4–2.2)
	High	15.0 (14.0–16.2)	6.6 (5.5–7.5)	6.3 (5.5–7.0)	6.4 (5.4–7.3)	6.1 (5.5–6.8)	6.0 (5.3–6.7)
**Juvenile survival (%)**	Low	—	2.7 (2.2–3.2)	2.7 (2.2–3.2)	1.1 (0.8–1.4)	2.7 (2.2–3.2)	1.0 (0.8–1.4)
	Moderate	—	6.0 (5.3–6.8)	6.0 (5.3–6.7)	2.3 (1.9–2.9)	6.0 (5.2–6.8)	2.4 (1.8–2.8)
	High	—	11.6 (10.6–12.7)	11.5 (10.5–12.6)	4.6 (4.0–5.3)	11.6 (10.6–12.7)	4.6 (4.0–5.2)

EPG, eggs per gram of feces; Moderate-to-heavy infection defined as infection prevalence with intensity ≥100 EPG.
Novel drug A assumes same efficacy against adult schistosomes as single-dose praziquantel, plus perfect efficacy against juvenile schistosomes.
Novel drug B assumes near perfect efficacy against adult schistosomes, with no activity against juvenile schistosomes.
Novel drug C assumes near perfect efficacy against adult schistosomes, plus perfect efficacy against juvenile schistosomes.
This low coverage setting could broadly represent settings where preventive chemotherapy is focused in school-age children alone, which represents a smaller proportion of the population being treated.

Table S3: Comparison of the impact of different anthelmintic drugs and dosing schedules after a single mass drug administration campaign with 90% coverage on *Schistosoma mansoni* prevalence.

	Setting	Baseline	1-dose praziquantel	2-dose praziquantel	Novel drug A	Novel drug B	Novel drug C
**Prevalence (%)**	Low	14.6 (13.4–15.6)	2.8 (1.6–3.8)	2.2 (1.6–2.8)	2.4 (1.2–3.4)	1.8 (1.4–2.2)	1.4 (1.2–1.8)
	Moderate	31.2 (29.8–32.6)	7.0 (4.2–8.6)	5.4 (4.0–6.6)	5.8 (3.2–7.6)	4.4 (3.8–5.0)	3.2 (2.8–3.6)
	High	52.8 (51.2–54.4)	14.0 (8.8–17.4)	10.8 (8.2–12.6)	11.4 (5.8–14.8)	8.6 (7.8–9.4)	5.4 (4.8–5.8)
**Moderate and heavy infection prevalence (%)**	Low	1.2 (0.8–1.6)	0.2 (0.0–0.2)	0.2 (0.0–0.2)	0.2 (0.0–0.2)	0.0 (0.0–0.2)	0.0 (0.0–0.2)
	Moderate	4.6 (3.8–5.2)	0.6 (0.2–1.0)	0.6 (0.2–0.8)	0.6 (0.2–0.8)	0.4 (0.2–0.6)	0.4 (0.2–0.6)
	High	15.0 (14.0–16.2)	2.2 (1.4–3.0)	1.8 (1.4–2.4)	2.0 (1.2–2.8)	1.6 (1.4–2.0)	1.4 (1.2–1.8)
**Juvenile survival (%)**	Low	—	2.7 (2.2–3.2)	2.6 (2.2–3.2)	0.2 (0.0–0.4)	2.7 (2.2–3.2)	0.2 (0.0–0.4)
	Moderate	—	6.0 (5.3–6.8)	6.0 (5.2–6.7)	0.6 (0.4–0.9)	6.0 (5.2–6.8)	0.6 (0.4–0.8)
	High	—	11.5 (10.6–12.8)	11.5 (10.6–12.6)	1.2 (0.8–1.5)	11.6 (10.6–12.7)	1.2 (0.8–1.4)

EPG, eggs per gram of feces; Moderate-to-heavy infection defined as infection prevalence with intensity ≥100 EPG.
Novel drug A assumes same efficacy against adult schistosomes as single-dose praziquantel, plus perfect efficacy against juvenile schistosomes.
Novel drug B assumes near perfect efficacy against adult schistosomes, with no activity against juvenile schistosomes.
Novel drug C assumes near perfect efficacy against adult schistosomes, plus perfect efficacy against juvenile schistosomes.

Table S4: Comparison of the impact of different anthelmintic drugs and dosing schedules after a single mass drug administration campaign with 100% coverage on *Schistosoma mansoni* prevalence.

	Setting	Baseline	1-dose praziquantel	2-dose praziquantel	Novel drug A	Novel drug B	Novel drug C
**Prevalence (%)**	Low	14.6 (13.4–15.6)	1.6 (0.4–2.4)	0.8 (0.4–1.4)	1.0 (0.0–2.0)	0.4 (0.2–0.7)	0.0 (0.0–0.0)
	Moderate	31.2 (29.8–32.6)	4.2 (1.4–6.2)	2.6 (1.2–3.6)	3.0 (0.4–4.8)	1.4 (1.0–1.8)	0.0 (0.0–0.2)
	High	52.8 (51.2–54.4)	10.0 (4.0–13.4)	6.2 (3.4–8.2)	7.0 (0.9–10.4)	3.6 (3.0–4.2)	0.2 (0.0–0.2)
**Moderate and heavy infection prevalence (%)**	Low	1.2 (0.8–1.6)	0.0 (0.0–0.2)	0.0 (0.0–0.0)	0.0 (0.0–0.0)	0.0 (0.0–0.0)	0.0 (0.0–0.0)
	Moderate	4.6 (3.8–5.2)	0.2 (0.0–0.4)	0.0 (0.0–0.2)	0.2 (0.0–0.4)	0.0 (0.0–0.0)	0.0 (0.0–0.0)
	High	15.0 (14.0–16.2)	0.8 (0.2–1.6)	0.4 (0.0–0.8)	0.6 (0.0–1.2)	0.2 (0.0–0.4)	0.0 (0.0–0.0)
**Juvenile survival (%)**	Low	—	2.7 (2.2–3.2)	2.7 (2.1–3.2)	0.0 (0.0–0.0)	2.7 (2.2–3.2)	0.0 (0.0–0.0)
	Moderate	—	6.0 (5.3–6.8)	5.9 (5.3–6.7)	0.0 (0.0–0.0)	6.0 (5.2–6.8)	0.0 (0.0–0.0)
	High	—	11.5 (10.6–12.7)	11.7 (10.6–12.6)	0.0 (0.0–0.0)	11.5 (10.7–12.7)	0.0 (0.0–0.0)

EPG, eggs per gram of feces; Moderate-to-heavy infection defined as infection prevalence with intensity ≥100 EPG.
Novel drug A assumes same efficacy against adult schistosomes as single-dose praziquantel, plus perfect efficacy against juvenile schistosomes.
Novel drug B assumes near perfect efficacy against adult schistosomes, with no activity against juvenile schistosomes.
Novel drug C assumes near perfect efficacy against adult schistosomes, plus perfect efficacy against juvenile schistosomes.
This model is not validated to estimate chances for elimination of transmission.

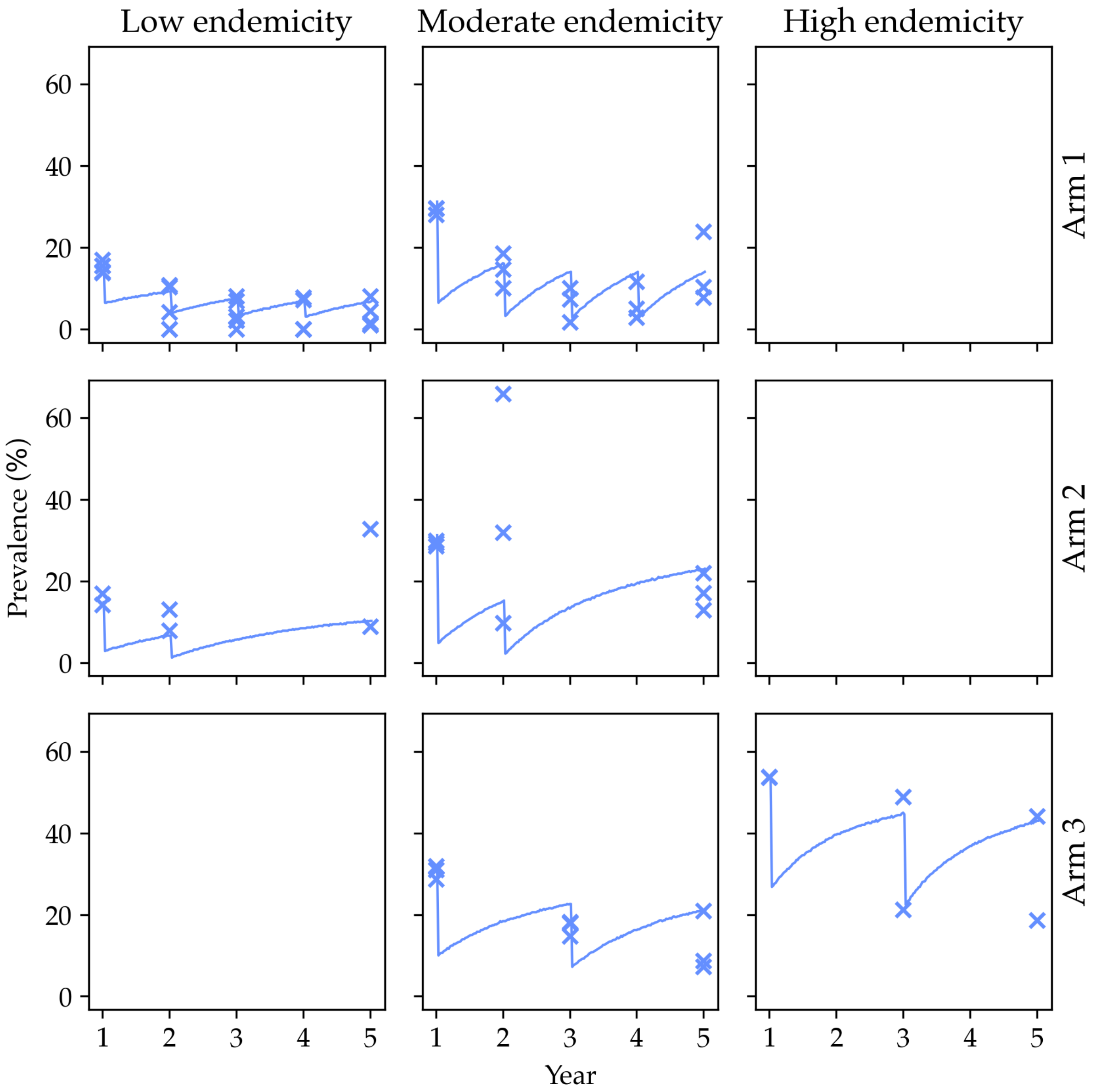

Figure S1: Model validation of long-term transmission dynamics with single dose praziquantel MDA in three epidemiologic settings. The line represents the model prediction while the crosses refer to observed data from the SCORE trials, which includes three study arms that determine number and timing of MDA. The simulations mostly fall within the range of reported prevalences for each year, endemicity, and study arm, providing a validation of our schistosomiasis transmission model over the long term.

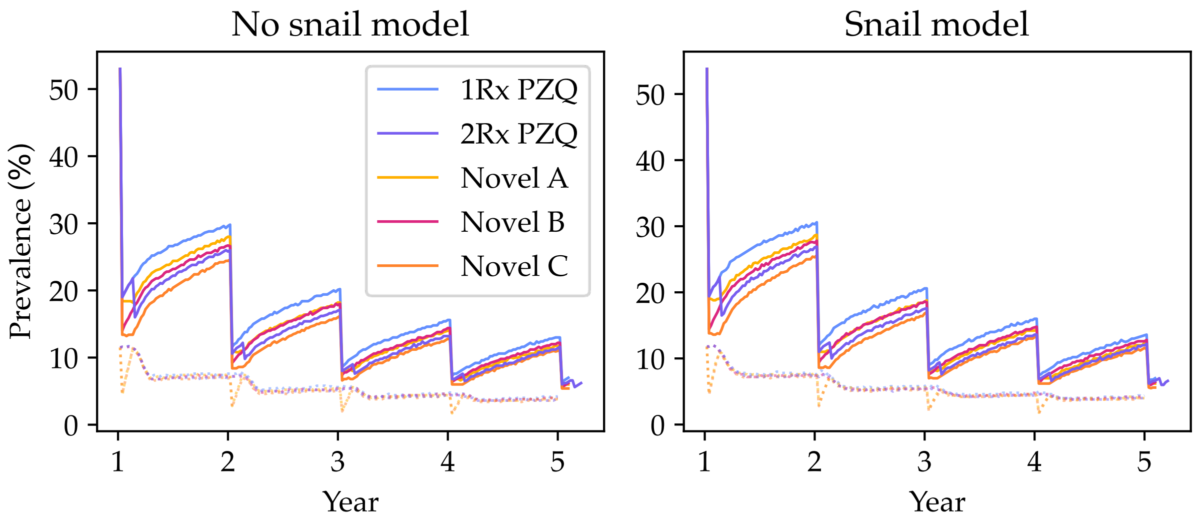

Figure S2: Sensitivity analysis using snail model. Comparison of the simulations of mass drug administration with different anthelmintic drugs and dosing schedules under 75% treatment coverage (with 10% systematic non-adherence), with force of infection determined in the left panel by the dynamic model as described in the main text and in the right panel by the dynamic model with a snail infection model. The snail model provides similar results to the simplified dynamic model, indicating that our dynamic model is a valid simplification of the highly complex environmental dynamics of schistosomiasis within the assumed conditions.

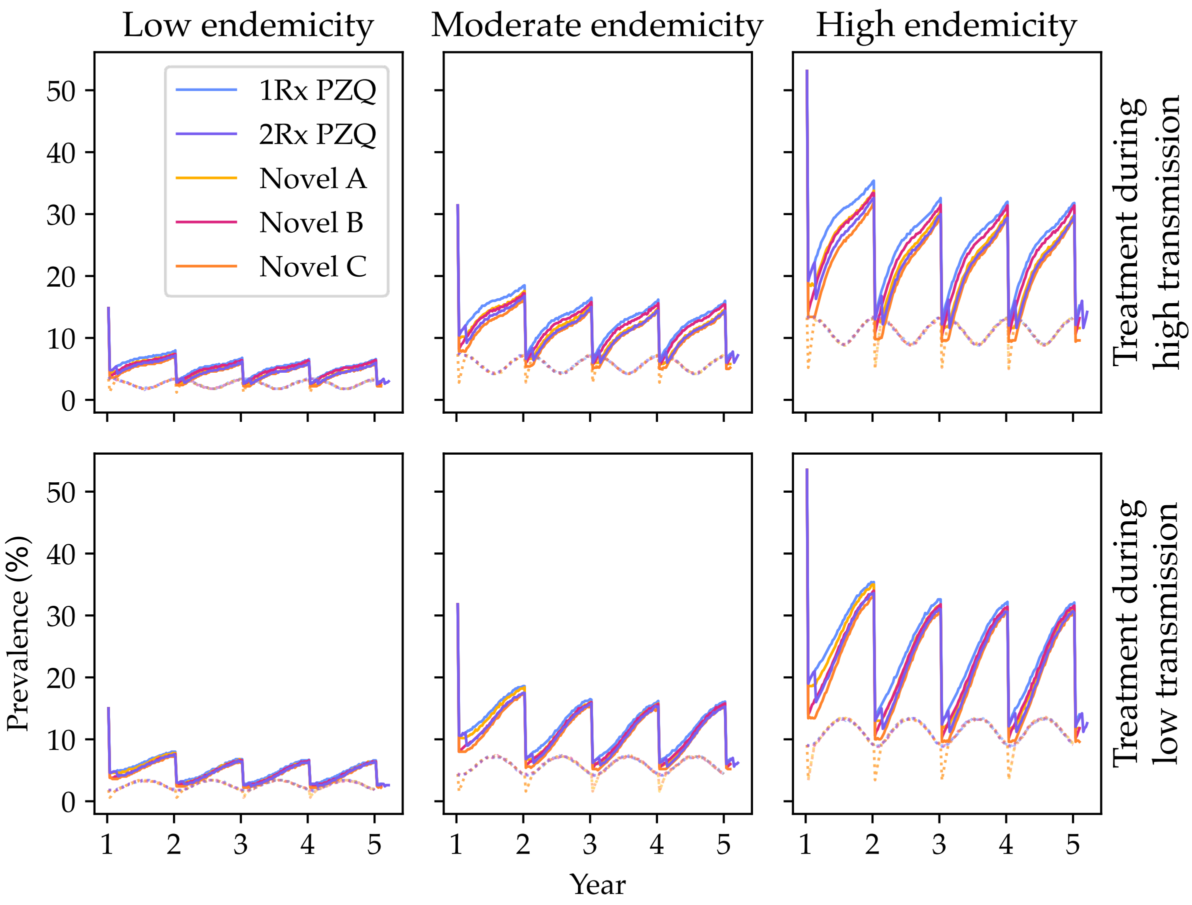

Figure S3: Sensitivity analysis using seasonal static model. Comparison of the impact of mass drug administration with different anthelmintic drugs and dosing schedules against schistosomiasis under 75% treatment coverage (with 10% systematic non-adherence), with a variant of the static model with seasonal transmission, ie, the force of infection varies with the sinusoid of time with a 1-year period. In the upper row, treatment is administered when the force of infection is at its maximum (high transmission season), and in the lower row treatment is administered when the force of infection is at its minimum (low transmission season).

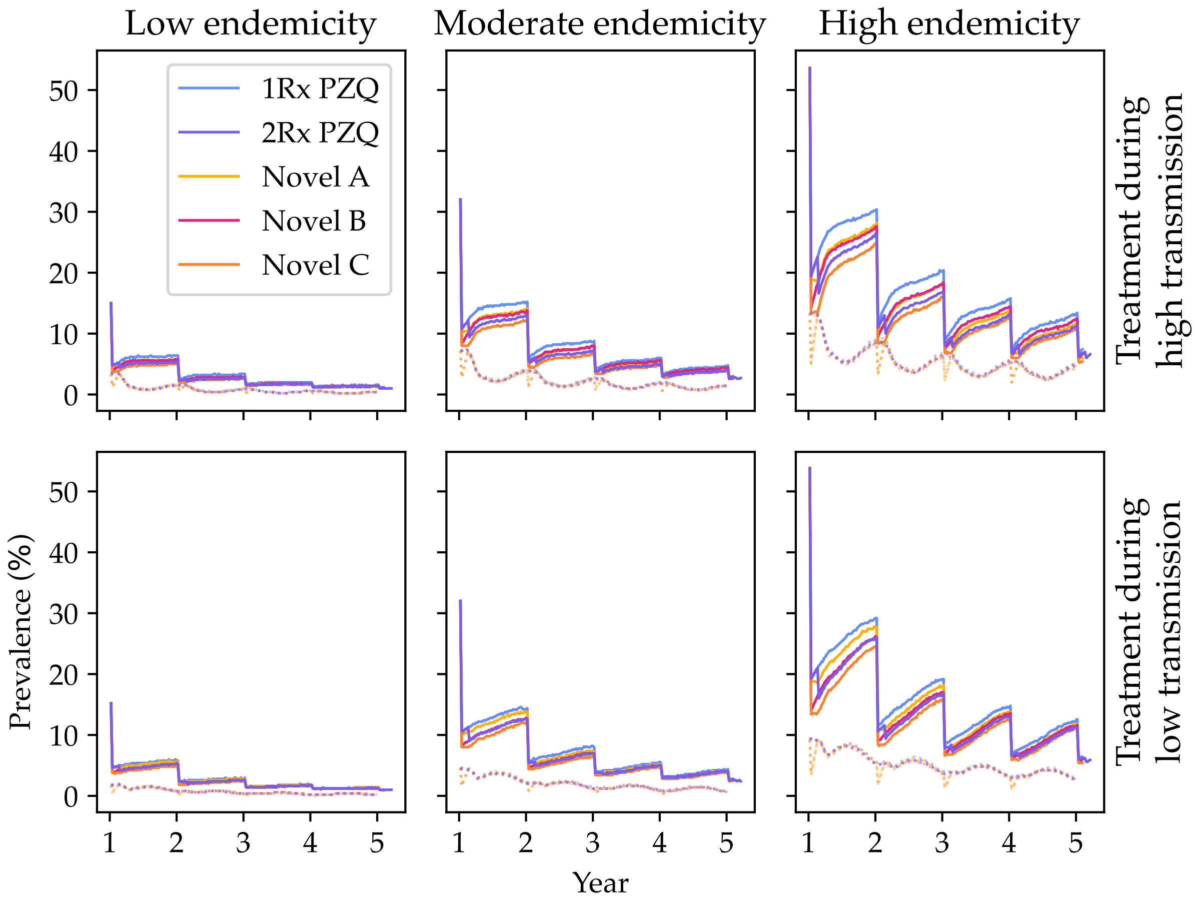

Figure S4: Sensitivity analysis using seasonal dynamic model. Comparison of the impact of mass drug administration with different anthelmintic drugs and dosing schedules against schistosomiasis under 75% treatment coverage (with 10% systematic non-adherence), with a variant of the dynamic model with seasonal transmission, ie, the force of infection varies with the sinusoid of time with a 1-year period. In the upper row, treatment is administered when the force of infection is at its maximum (high transmission season), and in the lower row treatment is administered when the force of infection is at its minimum (low transmission season).

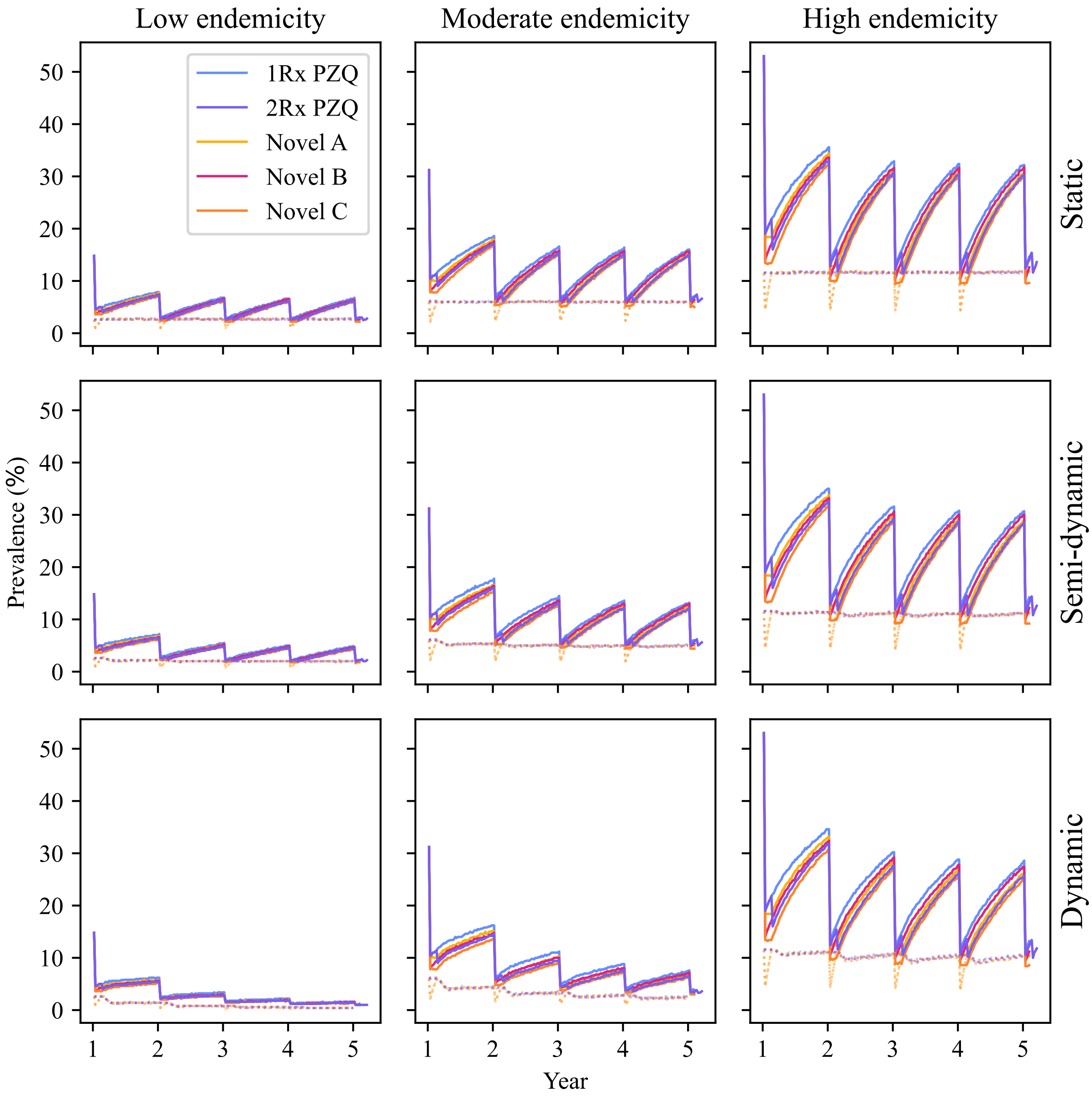

Figure S5: Sensitivity analysis using a dynamic model with saturation. Comparison of the impact of mass drug administration with different anthelmintic drugs and dosing schedules against schistosomiasis under 75% treatment coverage (with 10% systematic non-adherence), with a variant of the dynamic model with saturation, ie, force of infection is a non-linear function of population egg burden. The static model is the same as the base analysis and included for comparison.

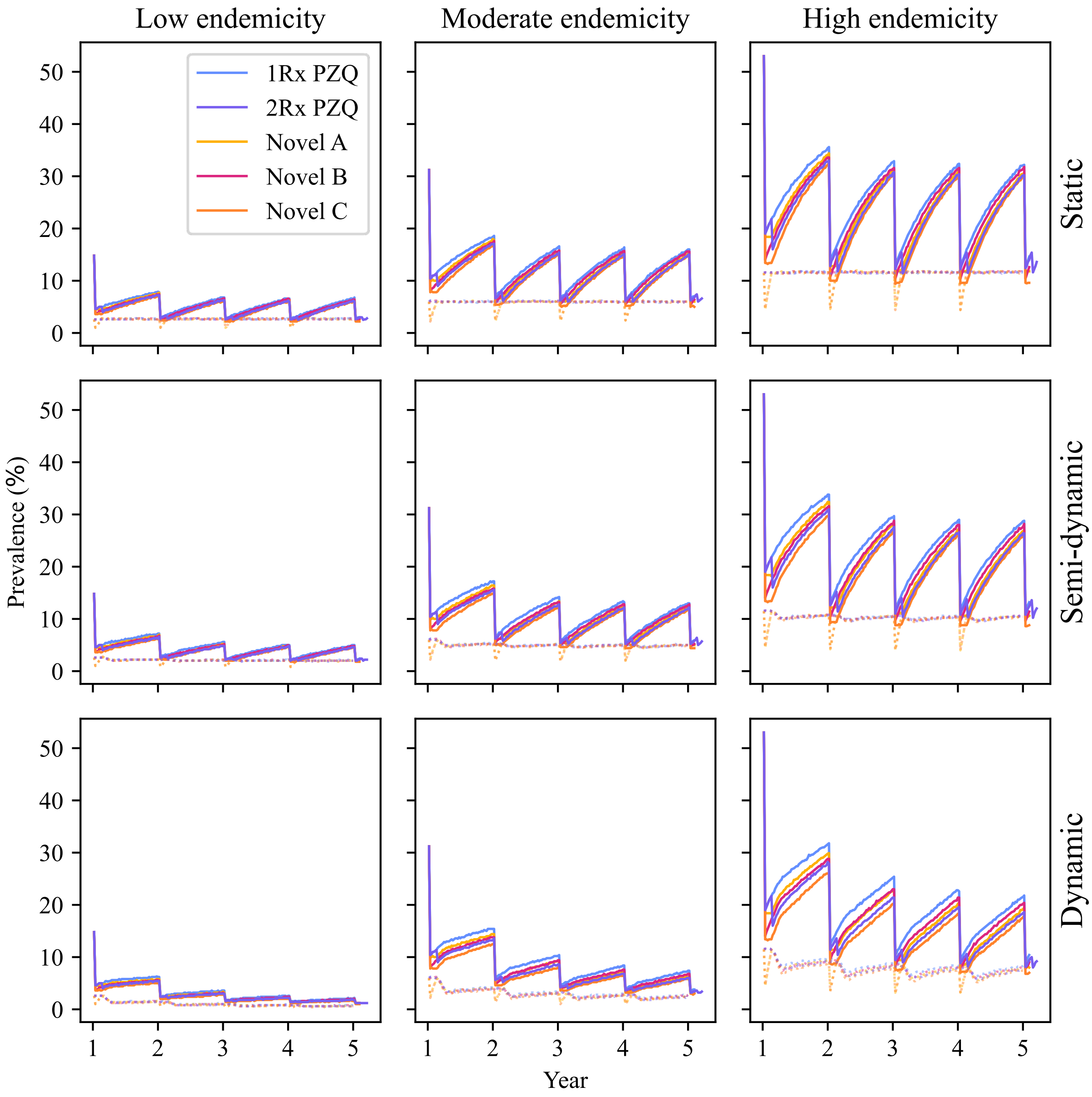

Figure S6: Sensitivity analysis using a dynamic model based on prevalence. Comparison of the impact of mass drug administration with different anthelmintic drugs and dosing schedules against schistosomiasis under 75% treatment coverage (with 10% systematic non-adherence), with a variant of the dynamic model assuming force of infection is proportional to prevalence of infection. The static model is the same as the base analysis and included for comparison.

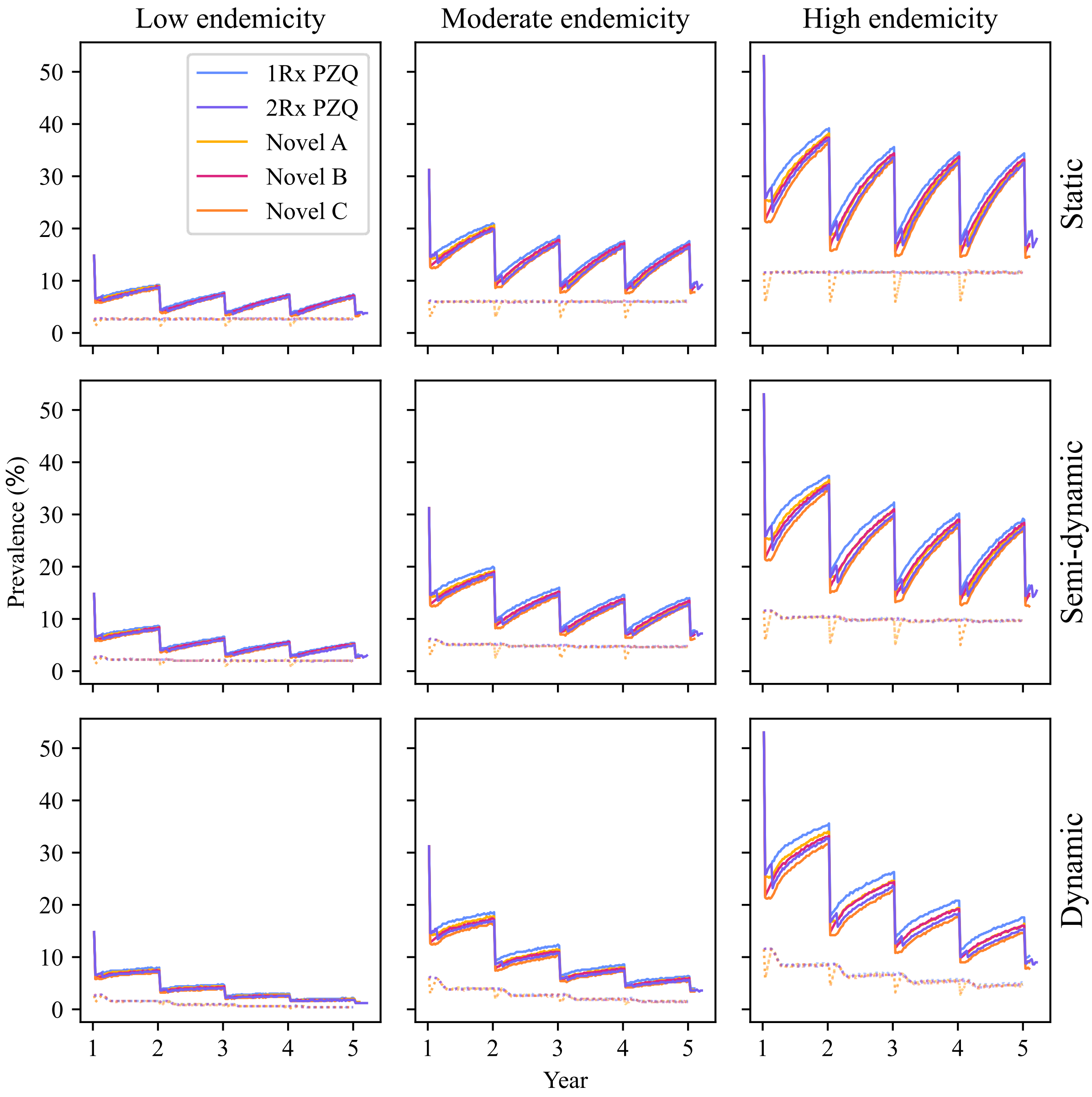

Figure S7: Sensitivity analysis with 60% coverage. Comparison of the impact of mass drug administration with different anthelmintic drugs and dosing schedules against schistosomiasis under 60% treatment coverage (with 10% systematic non-adherence) with different epidemiologic settings and transmission assumptions. This low coverage setting could broadly represent settings where preventive chemotherapy is focused in school-age children alone, which represents a smaller proportion of the population being treated.

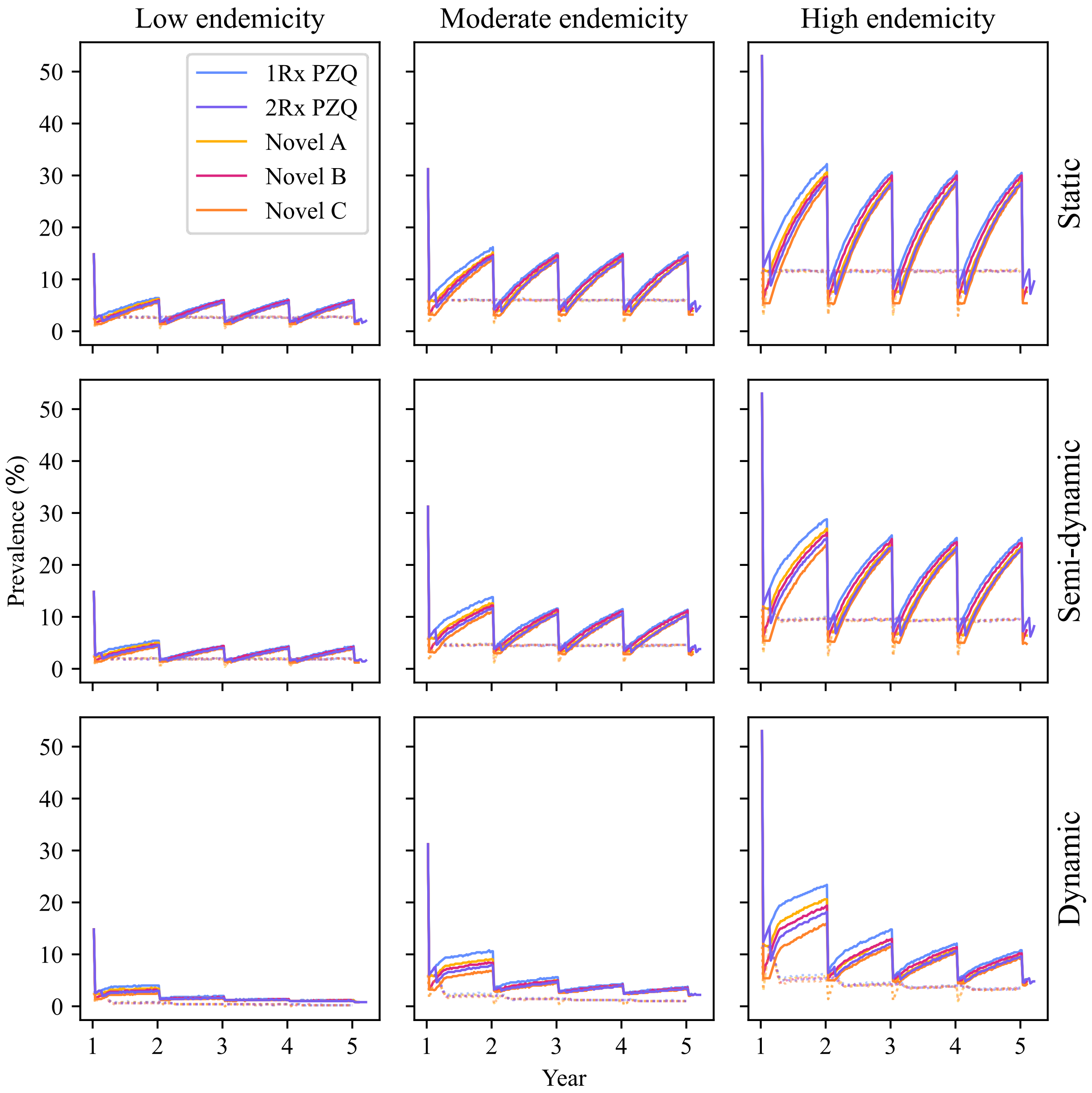

Figure S8: Sensitivity analysis with 90% coverage. Comparison of the impact of mass drug administration with different anthelmintic drugs and dosing schedules against schistosomiasis under 90% treatment coverage (with 10% systematic non-adherence) with different epidemiologic settings and transmission assumptions.

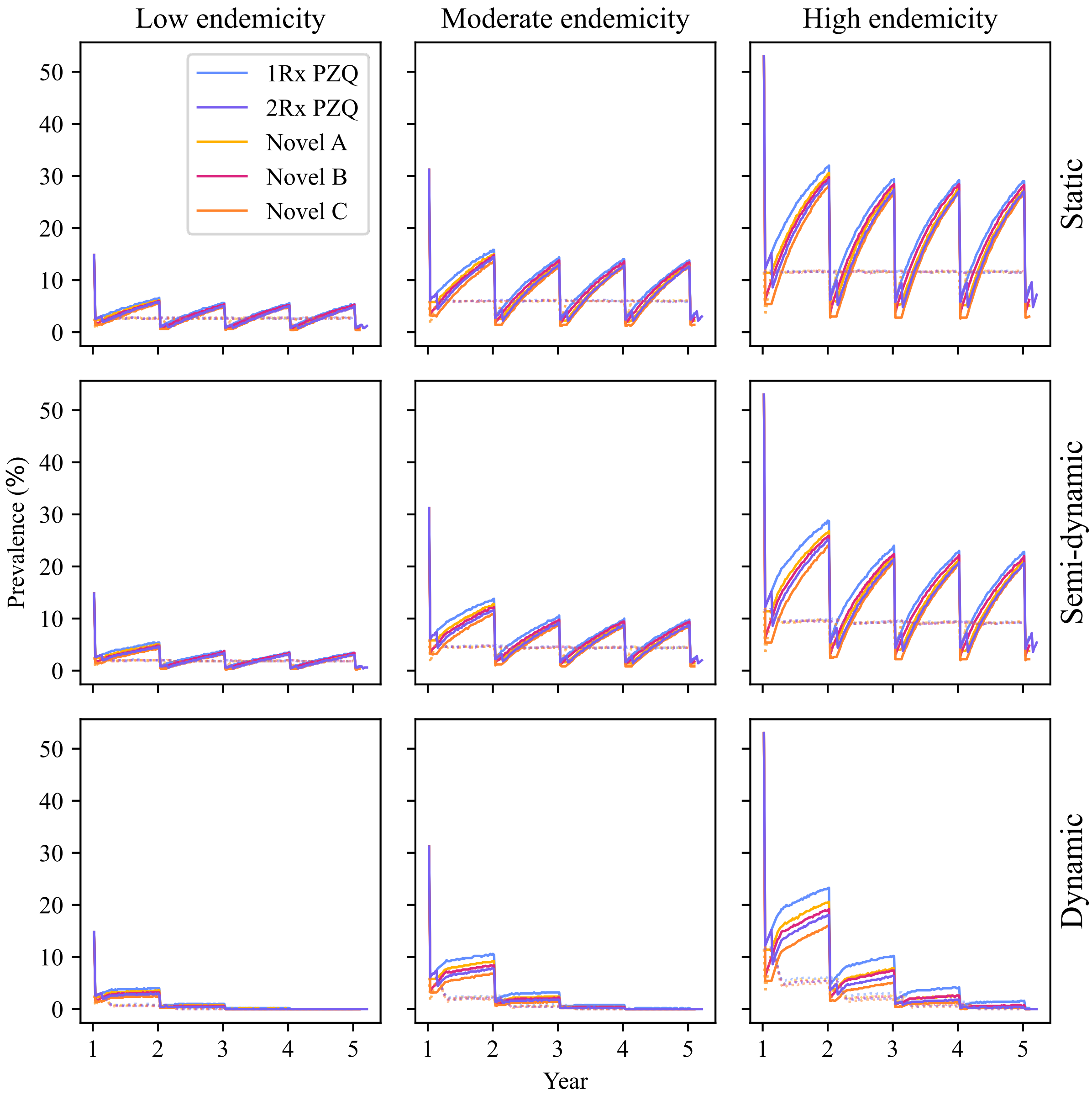

Figure S9: Sensitivity analysis with 90% coverage and 0% non-adherence. Comparison of the impact of mass drug administration with different anthelmintic drugs and dosing schedules against schistosomiasis under 90% treatment coverage (with 0% systematic non-adherence) with different epidemiologic settings and transmission assumptions. Note that this model has not been calibrated or validated to predict schistosomiasis elimination.

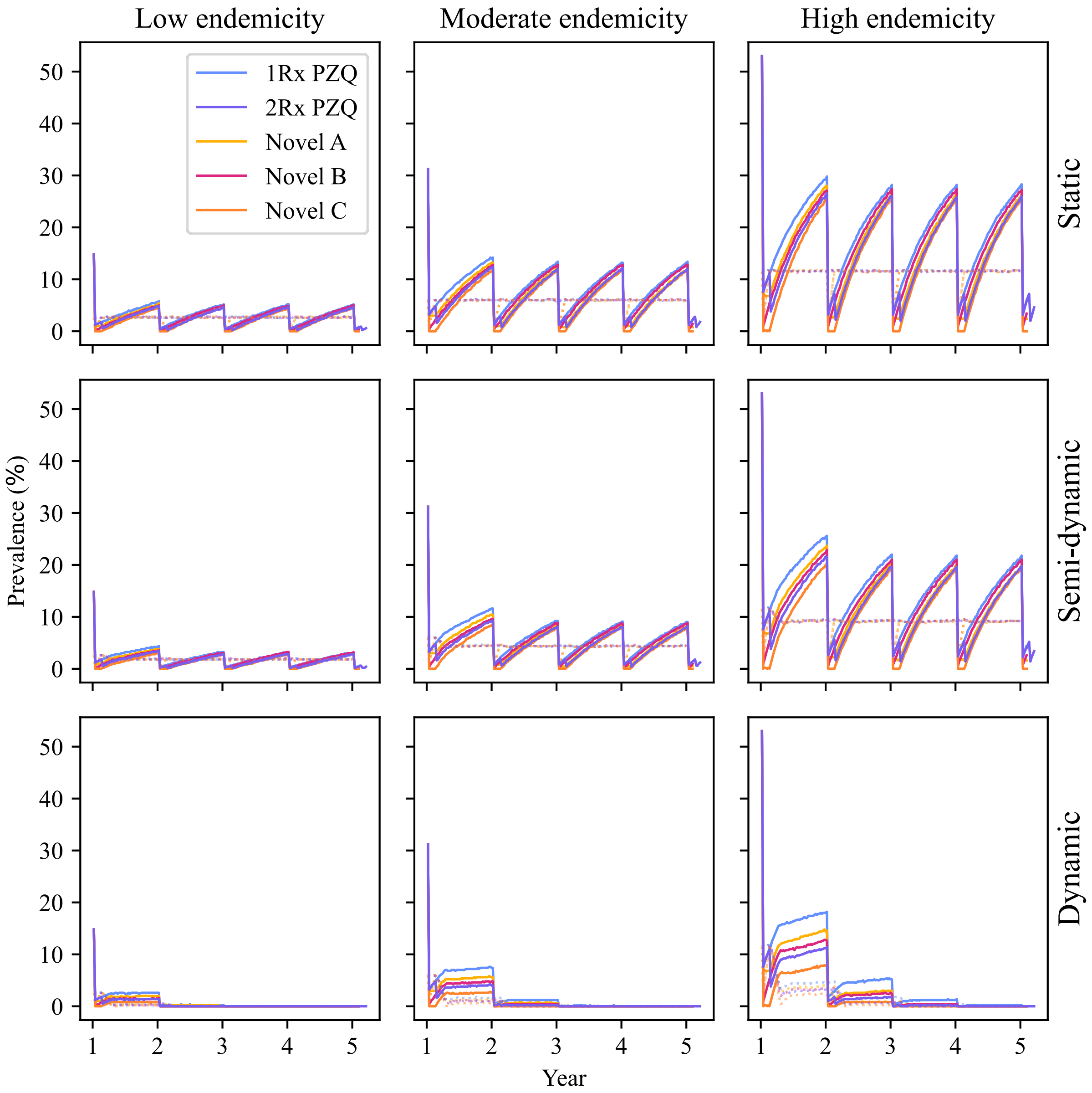

Figure S10: Sensitivity analysis with 100% coverage. Comparison of the impact of mass drug administration with different anthelmintic drugs and dosing schedules against schistosomiasis under 100% treatment coverage with different epidemiologic settings and transmission assumptions. Note that this model has not been calibrated or validated to predict schistosomiasis elimination.

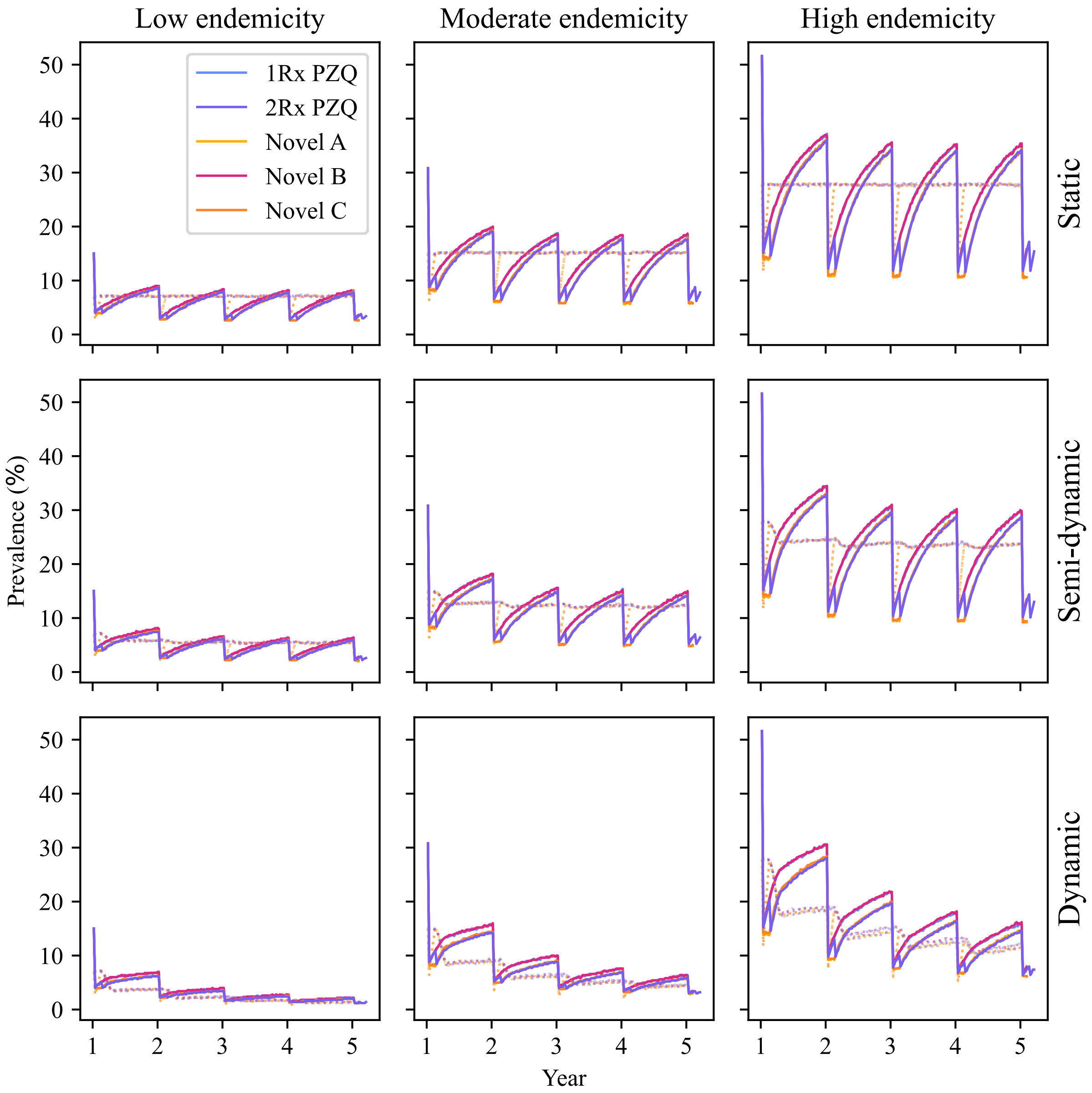

Figure S11: Sensitivity analysis with low schistosome fecundity. Comparison of the impact of mass drug administration with different anthelmintic drugs and dosing schedules against schistosomiasis, assuming that a single worm pair infection generates 1EPG of infection intensity (low schistosome fecundity scenario). Under this assumption, calibration to observed cure rates means that praziquantel is assumed to kill 99% of adult worms, meaning that the benefits of increased potency against adult worms are limited. To fit the baseline characteristics, this version of the model required that individuals were infected more often and had higher worm burden than in the main analysis.

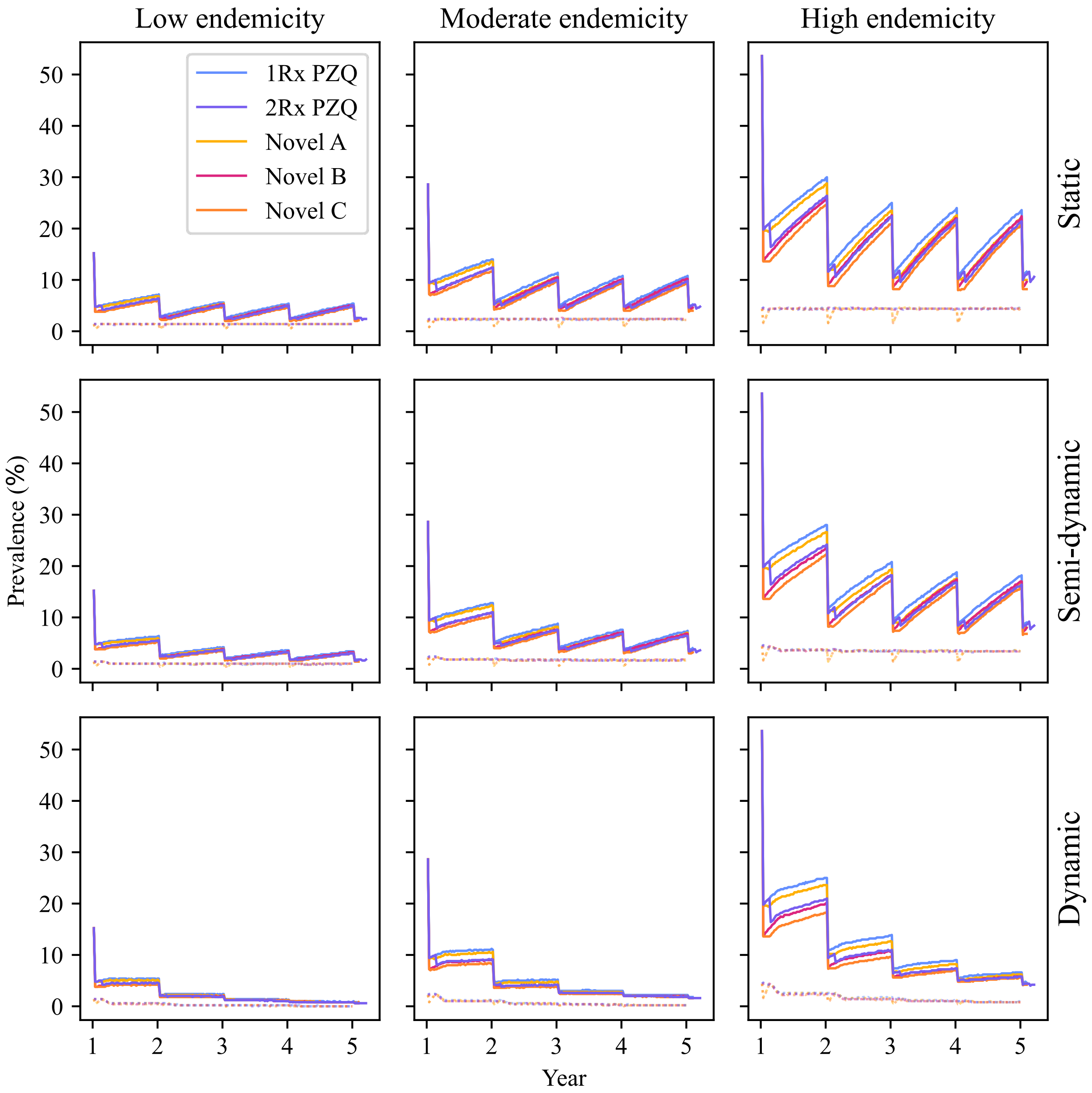

Figure S12: Sensitivity analysis with high schistosome fecundity. Comparison of the impact of mass drug administration with different anthelmintic drugs and dosing schedules against schistosomiasis, assuming that a single worm pair infection generates 15EPG of infection intensity (high schistosome fecundity scenario). Long term benefits of all treatment options are greater in this analysis. To fit the baseline characteristics, this version of the model required that individuals were infected less often and had lower worm burden than in the main analysis.

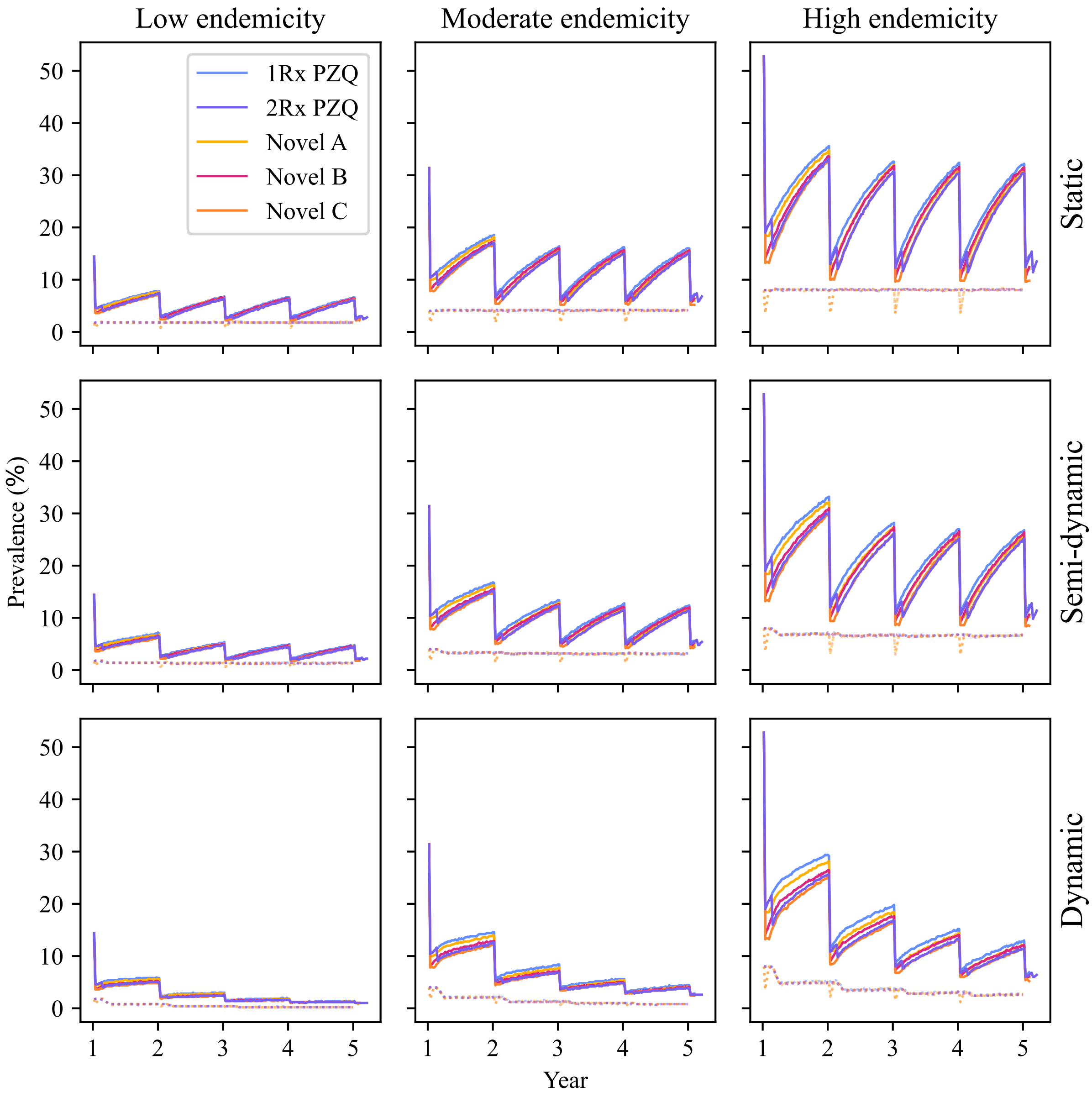

Figure S13: Sensitivity analysis with short juvenile life stage. Comparison of the impact of mass drug administration with different anthelmintic drugs and dosing schedules against schistosomiasis, assuming that the juvenile life stage lasts for 4 weeks.

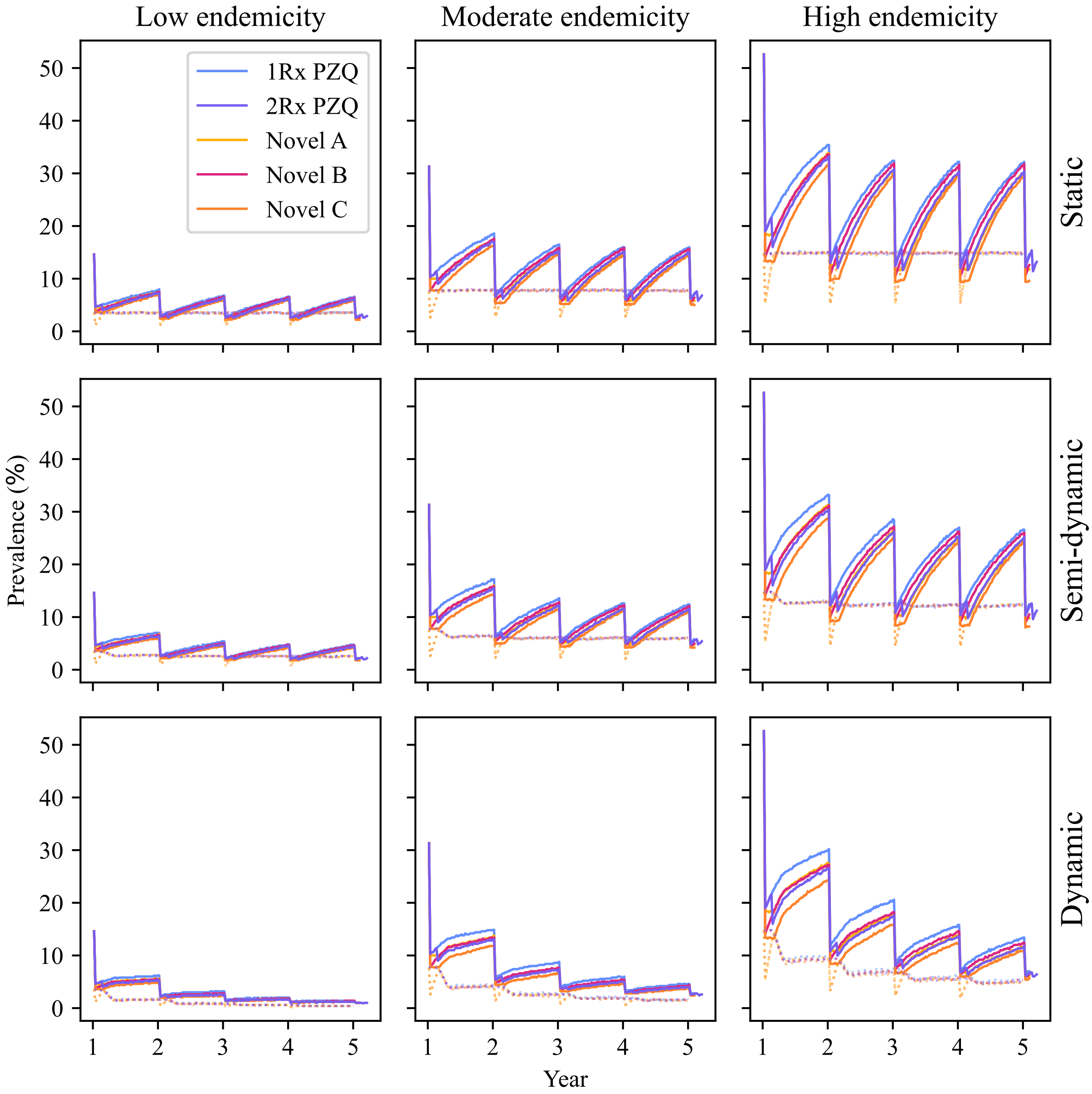

Figure S14: Sensitivity analysis with long juvenile life stage. Comparison of the impact of mass drug administration with different anthelmintic drugs and dosing schedules against schistosomiasis, assuming that the juvenile life stage lasts for 8 weeks.

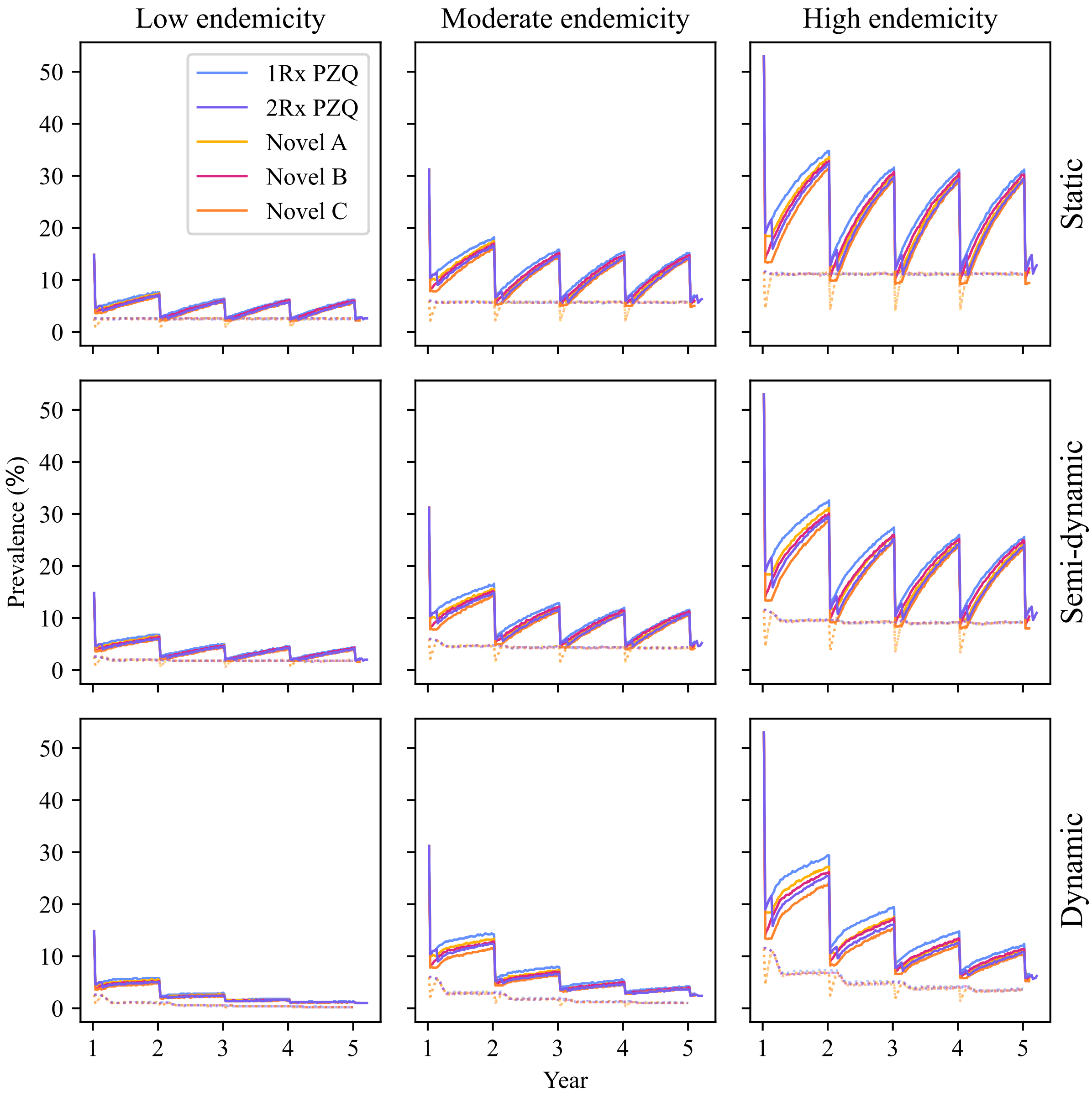

Figure S15: Sensitivity analysis with a WASH intervention. Comparison of the impact of mass drug administration with different anthelmintic drugs and dosing schedules against schistosomiasis, assuming that a water, sanitation, and hygiene (WASH) intervention is implemented alongside the first round of MDA which permanently reduces force of infection by 10%.

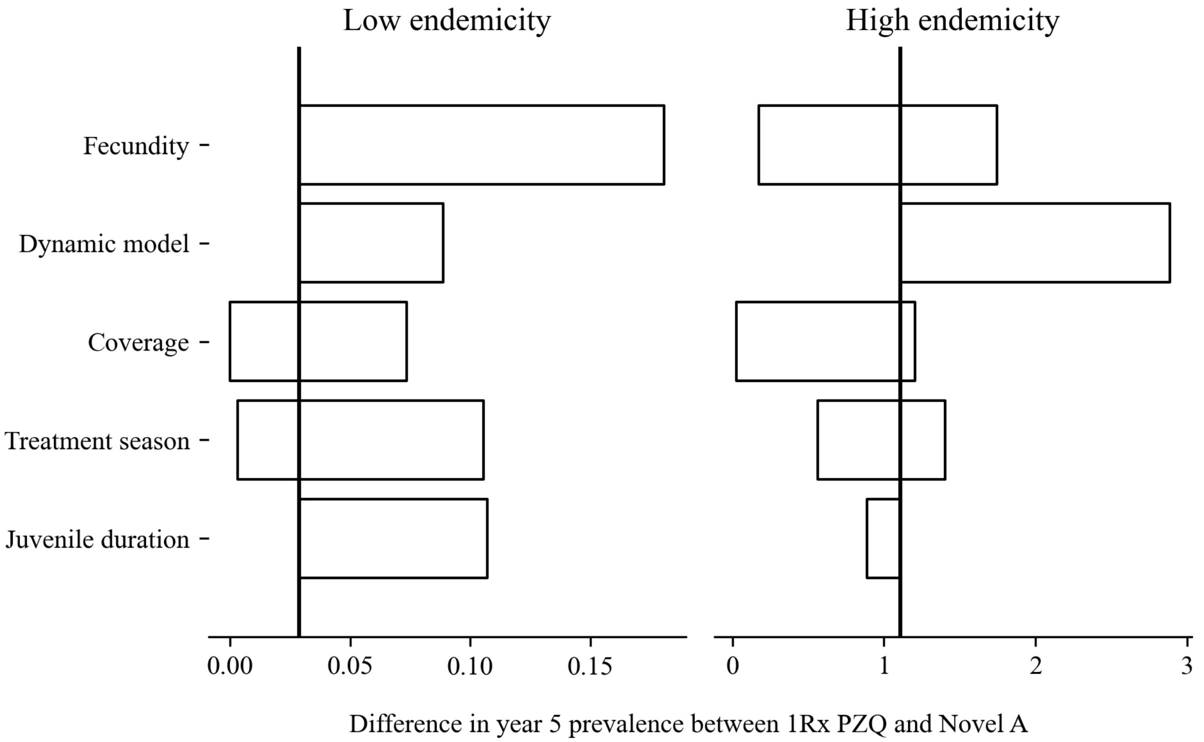

Figure S16: One-way sensitivity analysis on structural and parameter assumptions and their impact on the difference in impact of annual mass drug administration with single-dose praziquantel and novel drug A at year 5. We estimated the difference in prevalence between using single-dose praziquantel and novel drug A (praziquantel-equivalent activity against adult schistosomes plus perfect juvenile) in a 5-year mass drug administration program, varying a single structural or parameter assumption to estimate its impact on the model results. The vertical line represents the difference between single-dose praziquantel and novel drug A in the base case model. We varied: (i) assumptions in a dynamic transmission model by incorporating environmental saturation or prevalence dependence; (ii) worm fecundity (1-15 EPG per worm pair); (iii) coverage of mass drug administration (60-90%); (iv) accounting for seasonal transmission and treatment in high or low transmission season; and (v) juvenile schistosome duration (4-8 weeks). We estimated median *S. mansoni* infection prevalence after 5 years of mass drug administration in a low (left panel) and high (right panel) endemicity setting. Note the different scale of x-axis for the low and high endemicity setting.

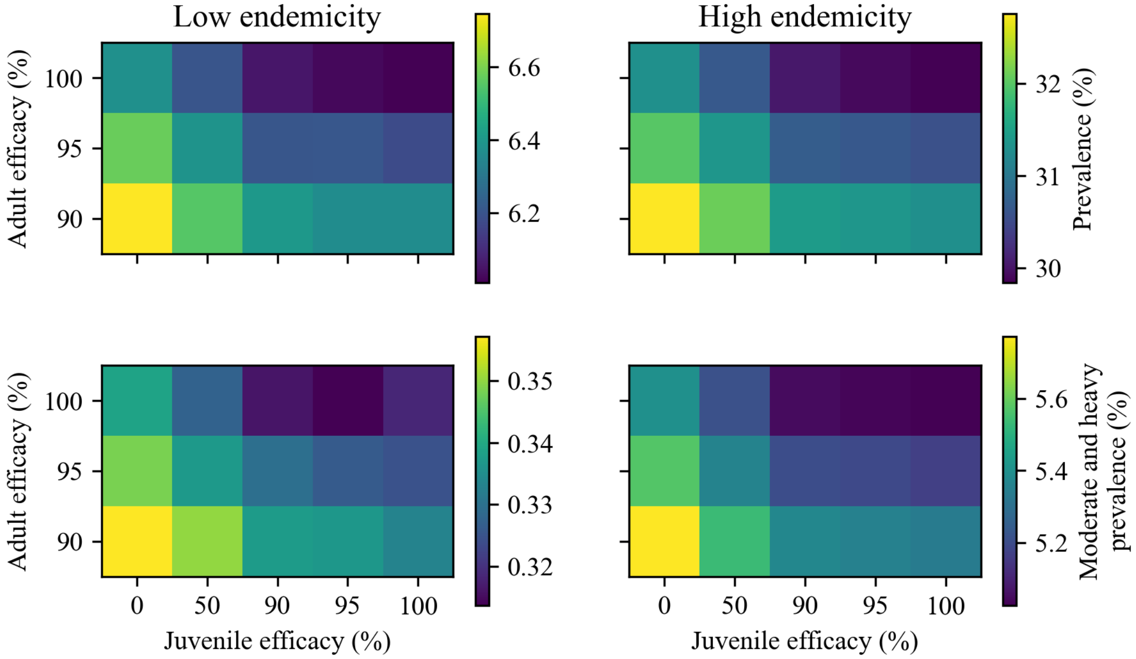

Figure S17: Year 5 static model outcomes before final mass drug administration with varying efficacy. Comparison of novel drug efficacy against adult and juvenile schistosomes on the overall population-level impact of annual mass drug administration at year 5, before the final round of mass drug administration, in the static model. We estimated mean *S. mansoni* infection prevalence (top) and prevalence of moderate and heavy infections (bottom).

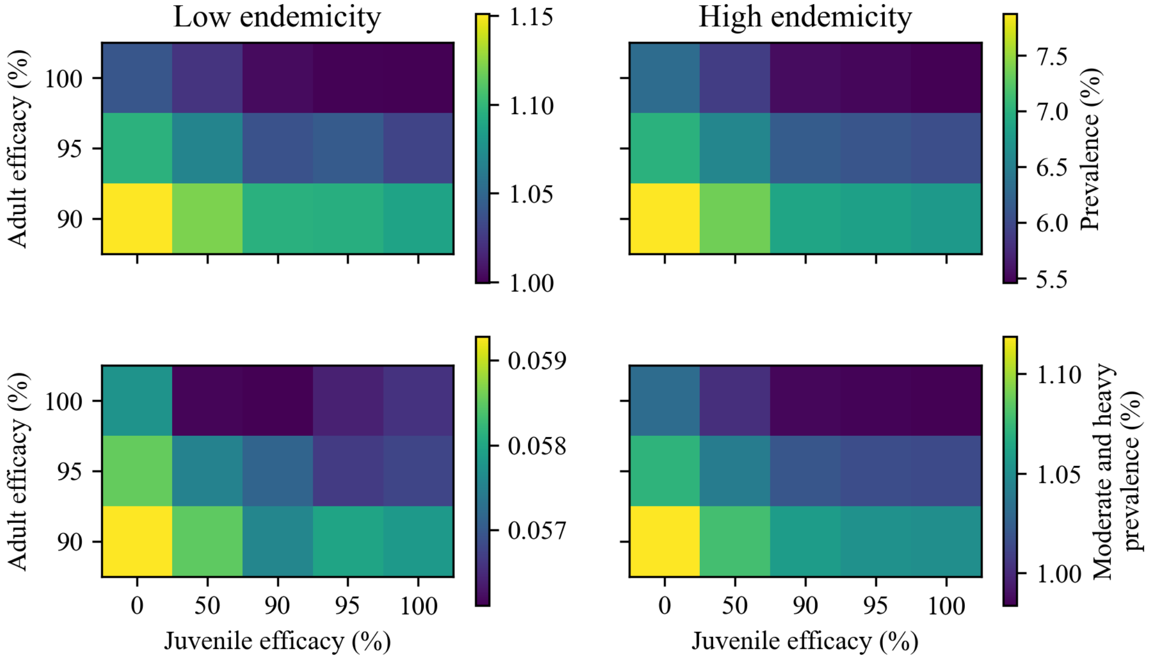

Figure S18: Year 5 dynamic model outcomes after final mass drug administration with varying efficacy. Comparison of novel drug efficacy against adult and juvenile schistosomes on the overall population-level impact of annual mass drug administration at year 5, after the final round of mass drug administration, in the dynamic model. We estimated mean *S. mansoni* infection prevalence (top) and prevalence of moderate and heavy infections (bottom).

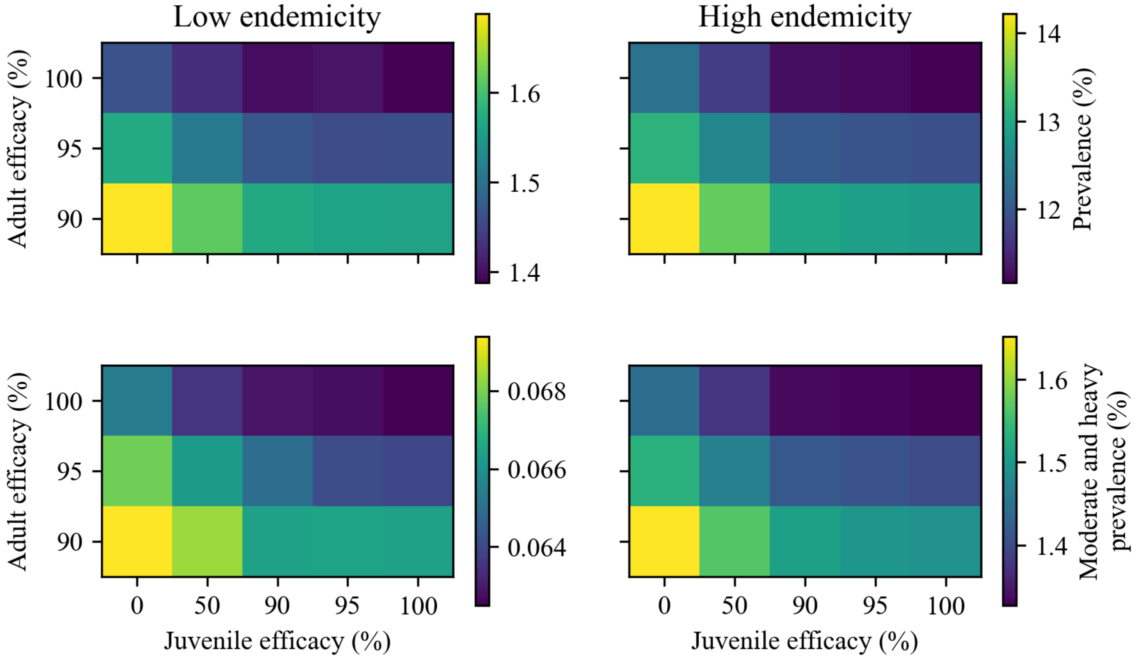

Figure S19: Year 5 dynamic model outcomes before final mass drug administration with varying efficacy. Comparison of novel drug efficacy against adult and juvenile schistosomes on the overall population-level impact of annual mass drug administration at year 5, before the final round of mass drug administration, in the dynamic model. We estimated mean *S. mansoni* infection prevalence (top) and prevalence of moderate and heavy infections (bottom).

References

1. Colley DG, Kittur N, Castleman JD, Karanja DMS, Mwinzi PNM, Kinung'hi SM, et al. ClinEpiDB. 2021 [cited 2022 Jul 20]. SCORE *S. mansoni* Cluster Randomized Trial. Available from: https://clinepidb.org/ce/app/workspace/analyses/DS_d6a1141fbf/new/details#Contacts

2. de Vlas S. Modelling human *Schistosoma mansoni* infection; the art of counting eggs in faeces [Doctoral dissertation - Doctoral dissertation]. [Rotterdam]: Erasmus Universiteit Rotterdam; 1996.

3. Moore DV, Sandground JH. The Relative Egg Producing Capacity of *Schistosoma mansoni* and *Schistosoma japonicum*. Am J Trop Med Hyg. 1956 Sep 1;5(5):831–40.

4. Cheever AW, Macedonia JG, Mosimann JE, Cheever EA. Kinetics of Egg Production and Egg Excretion by *Schistosoma mansoni* and *S. japonicum* in Mice Infected with a Single Pair of Worms. Am J Trop Med Hyg. 1994 Mar 1;50(3):281–95.

5. Bärenbold O, Garba A, Colley DG, Fleming FM, Haggag AA, Ramzy RMR, et al. Translating preventive chemotherapy prevalence thresholds for *Schistosoma mansoni* from the Kato-Katz technique into the point-of-care circulating cathodic antigen diagnostic test. PLoS Negl Trop Dis. 2018 Dec 14;12(12):e0006941.

6. Lo NC, Lai YS, Karagiannis-Voules DA, Bogoch II, Coulibaly JT, Bendavid E, et al. Assessment of global guidelines for preventive chemotherapy against schistosomiasis and soil-transmitted helminthiasis: a cost-effectiveness modelling study. Lancet Infect Dis. 2016 Sep 1;16(9):1065–75.

7. Ezeamama AE, He CL, Shen Y, Yin XP, Binder SC, Campbell CH, et al. Gaining and sustaining schistosomiasis control: study protocol and baseline data prior to different treatment strategies in five African countries. BMC Infect Dis. 2016 May 26;16(1):229.

8. Ruhamyankaka E, Brunk BP, Dorsey G, Harb OS, Helb DA, Judkins J, et al. ClinEpiDB: an open-access clinical epidemiology database resource encouraging online exploration of complex studies [Internet]. Gates Open Research; 2020 [cited 2022 Jul 20]. Available from: https://gatesopenresearch.org/articles/3-1661

9. King CH, Kittur N, Binder S, Campbell CH, N'Goran EK, Meite A, et al. Impact of Different Mass Drug Administration Strategies for Gaining and Sustaining Control of *Schistosoma mansoni* and *Schistosoma haematobium* Infection in Africa. Am J Trop Med Hyg. 2020 Jul;103(1_Suppl):14–23.

10. Assaré RK, Tian-Bi YNT, Yao PK, N'Guessan NA, Ouattara M, Yapi A, et al. Sustaining Control of Schistosomiasis Mansoni in Western Côte d'Ivoire: Results from a SCORE Study, One Year after Initial Praziquantel Administration. PLoS Negl Trop Dis. 2016 Jan 20;10(1):e0004329.

11. Navaratnam AMD, Sousa-Figueiredo JC, Stothard JR, Kabatereine NB, Fenwick A, Mutumba-Nakalembe MJ. Efficacy of praziquantel syrup versus crushed praziquantel tablets in the treatment of intestinal schistosomiasis in Ugandan preschool children, with observation on compliance and safety. Trans R Soc Trop Med Hyg. 2012 Jul 1;106(7):400–7.

12. Barakat R, Morshedy HE. Efficacy of two praziquantel treatments among primary school children in an area of high *Schistosoma mansoni* endemicity, Nile Delta, Egypt. Parasitology. 2011 Apr;138(4):440–6.

13. Obonyo CO, Muok EM, Mwinzi PN. Efficacy of artesunate with sulfalene plus pyrimethamine versus praziquantel for treatment of *Schistosoma mansoni* in Kenyan children: an open-label randomised controlled trial. Lancet Infect Dis. 2010 Sep 1;10(9):603–11.

14. Rabie I, El-Ghannam M, Engels D, Sabry H, Sayed H, Hassanein M, et al. Efficacy of mirazid in comparison with praziquantel in Egyptian *Schistosoma mansoni*-infected school children and households. Am J Trop Med Hyg. 2005 Feb 1;72(2):119–23.

15. Erko B, Degarege A, Tadesse K, Mathiwos A, Legesse M. Efficacy and side effects of praziquantel in the treatment of Schistosomiasis mansoni in schoolchildren in Shesha Kekele Elementary School, Wondo Genet, Southern Ethiopia. Asian Pac J Trop Biomed. 2012 Mar 1;2(3):235–9.

16. Barakat R, Abou El-Ela NE, Sharaf S, El Sagheer O, Selim S, Tallima H, et al. Efficacy and Safety of Arachidonic Acid for Treatment of School-Age Children in *Schistosoma mansoni* High-Endemicity Regions. Am J Trop Med Hyg. 2015 Apr 1;92(4):797–804.

17. Nalugwa A, Nuwaha F, Tukahebwa EM, Olsen A. Single Versus Double Dose Praziquantel Comparison on Efficacy and *Schistosoma mansoni* Re-Infection in Preschool-Age Children in Uganda: A Randomized Controlled Trial. PLoS Negl Trop Dis. 2015 May;9(5):e0003796.

18. Utzinger J, N'goran EK, N'dri A, Lengeler C, Tanner M. Efficacy of praziquantel against *Schistosoma mansoni* with particular consideration for intensity of infection. Trop Med Int Health. 2000;5(11):771–8.

19. Munisi DZ, Buza J, Mpolya EA, Angelo T, Kinung'hi SM. The Efficacy of Single-Dose versus Double-Dose Praziquantel Treatments on *Schistosoma mansoni* Infections: Its Implication on Undernutrition and Anaemia among Primary Schoolchildren in Two On-Shore Communities, Northwestern Tanzania. BioMed Res Int. 2017 Sep 28;2017:e7035025.

20. Zwang J, Olliaro P. Efficacy and safety of praziquantel 40 mg/kg in preschool-aged and school-aged children: a meta-analysis. Parasit Vectors. 2017 Jan 26;10(1):47.

21. King CH, Olbrych SK, Soon M, Singer ME, Carter J, Colley DG. Utility of Repeated Praziquantel Dosing in the Treatment of Schistosomiasis in High-Risk Communities in Africa: A Systematic Review. PLoS Negl Trop Dis. 2011 Sep 20;5(9):e1321.

22. Saeed MEM, Krishna S, Greten HJ, Kremsner PG, Efferth T. Antischistosomal activity of artemisinin derivatives in vivo and in patients. Pharmacol Res. 2016 Aug;110:216–26.

23. Pérez del Villar L, Burguillo FJ, López-Abán J, Muro A. Systematic Review and Meta-Analysis of Artemisinin Based Therapies for the Treatment and Prevention of Schistosomiasis. PLoS ONE. 2012 Sep 21;7(9):e45867.

24. Liu R, Dong HF, Guo Y, Zhao QP, Jiang MS. Efficacy of praziquantel and artemisinin derivatives for the treatment and prevention of human schistosomiasis: a systematic review and meta-analysis. Parasit Vectors. 2011 Oct 17;4:201.

25. Mnkugwe RH, Minzi O, Kinung'hi S, Kamuhabwa A, Aklillu E. Efficacy and safety of praziquantel and dihydroartemisinin piperaquine combination for treatment and control of intestinal schistosomiasis: A randomized, non-inferiority clinical trial. PLoS Negl Trop Dis. 2020 Sep 23;14(9):e0008619.

26. De Clercq D, Vercruysse J, Kongs A, Verlé P, Dompnier JP, Faye PC. Efficacy of artesunate and praziquantel in *Schistosoma haematobium* infected schoolchildren. Acta Trop. 2002 Apr 1;82(1):61–6.

27. Civitello DJ, Angelo T, Nguyen KH, Hartman RB, Starkloff NC, Mahalila MP, et al. Transmission potential of human schistosomes can be driven by resource competition among snail intermediate hosts. Proc Natl Acad Sci U S A. 2022 Feb 8;119(6):e2116512119.

28. Malizia V, Vlas SJ de, Roes KCB, Giardina F. Revisiting the impact of *Schistosoma mansoni* regulating mechanisms on transmission dynamics using SchiSTOP, a novel modelling framework [Internet]. medRxiv; 2024 [cited 2024 Apr 25]. p. 2024.02.16.24302940. Available from: https://www.medrxiv.org/content/10.1101/2024.02.16.24302940v1
